# Enzymatic *N*-Allylation of Primary and Secondary Amines Using Renewable Cinnamic Acids Enabled by Bacterial Reductive Aminases

**DOI:** 10.1021/acssuschemeng.2c01180

**Published:** 2022-05-06

**Authors:** Godwin A. Aleku, Gabriel R. Titchiner, George W. Roberts, Sasha R. Derrington, James R. Marshall, Florian Hollfelder, Nicholas J. Turner, David Leys

**Affiliations:** † Manchester Institute of Biotechnology, Department of Chemistry, University of Manchester, Manchester, 131 Princess Street, Manchester M1 7DN, U.K.; ‡ Department of Biochemistry, University of Cambridge, 80 Tennis Court Road, Cambridge CB2 1GA, U.K.

**Keywords:** biocatalysis, biocatalytic reductive amination, biocatalytic cascades, reductive aminases, carboxylic acid reductases, allylic amines

## Abstract

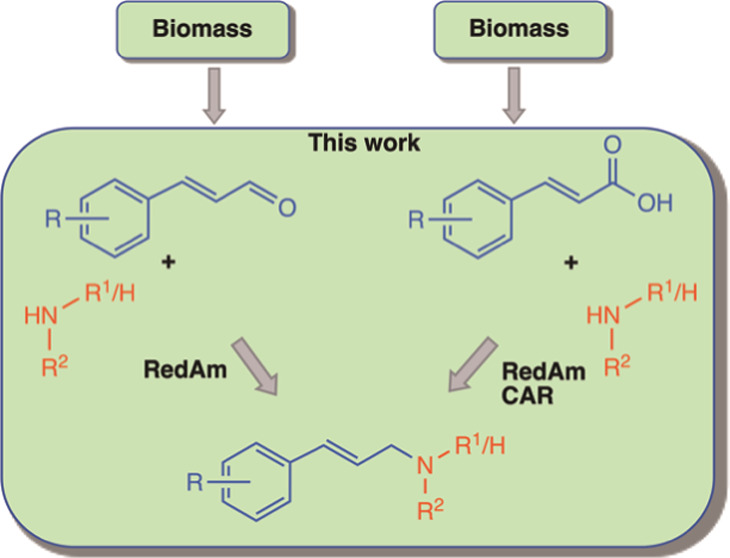

Allylic amines are a versatile class of synthetic precursors of many valuable nitrogen-containing organic compounds, including pharmaceuticals. Enzymatic allylic amination methods provide a sustainable route to these compounds but are often restricted to allylic primary amines. We report a biocatalytic system for the reductive *N*-allylation of primary and secondary amines, using biomass-derivable cinnamic acids. The two-step one-pot system comprises an initial carboxylate reduction step catalyzed by a carboxylic acid reductase to generate the corresponding α,β-unsaturated aldehyde *in situ*. This is followed by reductive amination of the aldehyde catalyzed by a bacterial reductive aminase pIR23 or BacRedAm to yield the corresponding allylic amine. We exploited pIR23, a prototype bacterial reductive aminase, self-sufficient in catalyzing formal reductive amination of α,β-unsaturated aldehydes with various amines, generating a broad range of secondary and tertiary amines accessed in up to 94% conversion under mild reaction conditions. Analysis of products isolated from preparative reactions demonstrated that only selective hydrogenation of the C=N bond had occurred, preserving the adjacent alkene moiety. This process represents an environmentally benign and sustainable approach for the synthesis of secondary and tertiary allylic amine frameworks, using renewable allylating reagents and avoiding harsh reaction conditions. The selectivity of the system ensures that bis-allylation of the alkylamines and (over)reduction of the alkene moiety are avoided.

## Abstract 2

A biocatalytic route for the *N*-allylation of primary and secondary amines using renewable cinnamic acids has been developed.

## Introduction

**Figure 1:**
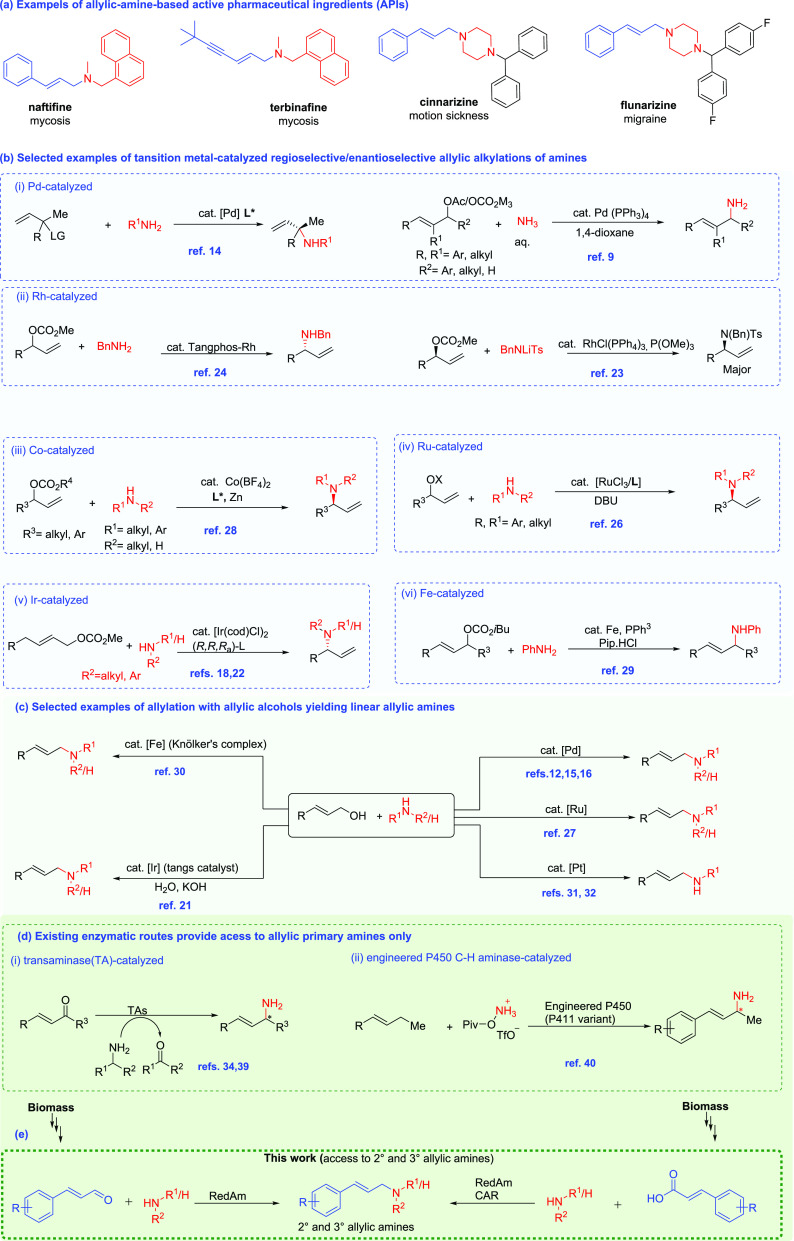
Overview of existing methods for the *N*-allylation of alkylamines. (a) Examples of allylic amine-containing pharmaceuticals; (b) selected examples of transition-metal-catalyzed *N*-allylic alkylation using activated allylic substrates; (c) selected examples of transition-metal-catalyzed *N*-allylation of alkylamines for the synthesis of linear allylic amines using allylic alcohols as allyl sources; (d) existing enzymatic routes for the synthesis of allylic primary amines; and (e) proposed enzymatic routes to accessing secondary and tertiary allylic amines described in this work. RedAm, reductive aminase; CAR, carboxylic acid reductase.

Allylic amines are a versatile class of synthetic building blocks frequently used to construct valuable nitrogen-containing organic skeletons, including bioactive medicinal compounds.^1,2^ As such, they often feature in many therapeutic drug classes, for example, antifungals (terbinafine, naftifine),^2^ antihistamines (cinnarizine),^3^ calcium channel blockers (flunarizine),^4^ and antidepressants (zimelidine)^5^ (Figure 1a). In addition, the reactivity of the allylic moiety allows the installation of other functional groups,^6,7^ which can be exploited as a site to fine-tune drug properties as performed in structure–activity relationship (SAR) studies. In view of the versatility of these scaffolds, a variety of chemo-catalytic synthesis methods have been developed.^8^ Of these approaches, transition-metal-catalyzed allylic substitution reactions are among the most widely employed methods for the *N*-allylation of alkylamines and ammonia.^8−11^ Initial efforts focused on Pd-catalyzed allylic substitution reactions (Tsuji–Trost reactions),^1^ and several Pd-complexes have been developed and explored for the synthesis of allylic amines.^9,12−17^ Progress in this area over the last decades has resulted in a significantly expanded library of metal-catalysts/ligands (complexes of Pd,^9,12−17^ Ir,^10,18−22^ Rh,^11,23−25^ Ru,^26,27^ Co,^28^ Fe,^29,30^ and Pt^31,32^), providing efficient *N*-allylation routes to simple linear as well as branched allylic amine products (Figure 1). In many cases, these transformations, especially those catalyzed by Ir-, Rh-, and Co- complexes, often result in branched, chiral products (Figure 1b).^1,8,10,25,26,28^

Despite the enormous achievements in this field, efficient, sustainable, and environmentally friendly methods that allow selective *N*-allylic monoalkylation of alkylamines using greener allylating reagents are still highly sought-after. More so that the frequently employed allylating reagents in these transformations such as carbonates and acetates generate stoichiometric quantities of wastes. As a consequence, recent efforts have favored the use of greener reagents such as allylic alcohols,^33^ with several groups demonstrating the direct use of allylic alcohols as allylating substrates with/without Lewis acid activators (Figure 1c).^12,21,27,30,31,34,35^

α,β-Unsaturated carboxylic acids such as cinnamic acid derivatives are renewable, biomass-derivable, and readily accessible allylating materials, as such represent green alternative alkylating agents. The direct *N*-allylation of amines with renewable cinnamic acids not only minimizes unwanted byproducts but can also shorten a synthetic route to a target allylic amine product. In spite of these advantages, to our knowledge, the direct use of a broad range of α,β-unsaturated carboxylic acids as versatile renewable starting materials for *N*-allylation of alkylamines has yet to be investigated.

Given the green credentials and high selectivity of biocatalytic approaches,^36−38^ there is currently great interest in developing sustainable and environmentally friendly enzymatic allylic amination methods to support green manufacturing of allylic amine building blocks. This interest has led to the investigation of enzymatic allylic amination routes (Figure 1d), using transaminases (TA)^34,39^ or an engineered cytochrome P450 (P411) C–H aminase.^40^ However, a notable limitation of both systems is the inherently restricted access to allylic primary amines only, highlighting an important need for alternative biocatalytic methods that can provide direct and selective access to monoallylated secondary and tertiary allylic amines.

To address these gaps, we sought to develop an efficient biocatalytic route to these amine scaffolds from readily available biomass-derivable and versatile starting materials (*e.g*., cinnamaldehydes, cinnamic acids) (Figure 1e). Building on our recent work demonstrating access to allylic alcohols from acrylic acids,^41^ and two recent reports of enzymatic methods for the amination of saturated carboxylic acids,^42,43^ we aimed at developing a one-pot system for the reductive *N-*allylation of primary and secondary amines using biomass-derivable acrylic acids. Our strategy seeks to link the well-established enzymatic carboxylate reduction^44,45^ to a novel biocatalytic reductive amination of α,β-unsaturated aldehydes with simple primary and secondary amines. In this way, a broad range of allylic secondary and tertiary amines can be generated.

## Results and Discussion

### Identification of Bacterial Reductive Aminases for the Amination of α,β-Unsaturated Aldehydes

First, we set out to identify a suitable aminating enzyme for the reductive amination step. The synthetic utility of imine reductases (IREDs) and reductive aminases (RedAms) in the synthesis of secondary amines is well documented.^46−50^ However, their suitability for the amination of α,β-unsaturated aldehydes to generate the corresponding allylic analogues is yet to be investigated. In catalyzing this desirable transformation, the sought-after biocatalyst must be efficient in overcoming the additional stability of the conjugated imines (*vs* nonconjugated imines) while also being highly chemoselective in exclusively reacting with the C=N functionality of the substrates and being inert to the adjacent C=C bond. The latter feature is particularly important in the light of a recent discovery that a subset of IREDs can catalyze conjugate reduction–reductive amination of enones.^51^

To utilize readily available and biomass-derivable versatile starting materials such as cinnamaldehyde and cinnamic acid derivatives as allylating agents, we assayed the recently described 384 metagenomic IRED collection developed by the Turner group and Prozomix^52^ to identify enzyme candidates acting on these substrates. A preliminary colorimetric lysate-based assay monitoring the oxidative deamination of *N*-cinnamylcyclopropanamine **2a** returned five active hits, previously described as pIR13, pIR23, pIR107, pIR114, and pIR120.^52^

Upon a secondary gas chromatographic (GC)-based biotransformation analysis of the five variants but monitoring the desired forward reaction (*i.e*., reductive amination of cinnamaldehyde **2** with cyclopropylamine **a**) revealed pIR23 (*Cystobacter ferrugineus* reductive aminase) as the best performing enzyme (Supporting Information, Figure S1).

Given our interest in the mechanistic aspects of bacterial IRED-catalyzed reductive amination, we also recruited and characterized a novel RedAm-like protein from a bacterium (*Bac*RedAm) isolated from a compost metagenome (Zoo Composter 4, Sao Paulo Zoo, Brazil). Sequence analysis comparing the well-characterized fungal RedAms (*Asp*RedAm and *Ad*RedAm) against homologous bacterial sequences available in GenBank (as of September 2020) revealed *Bac*RedAm as the most homologous bacterial sequence (GenBank ref: PZN88780.1; >50% sequence identity to *Asp*RedAm, *Ad*RedAm). More so, the active site residues of fungal RedAms are conserved in *Bac*RedAm. Indeed, *Bac*RedAm displayed similar kinetic parameters and amine substrate scope to *Asp*RedAm and *Ad*RedAm (Figure S2), making it a prototype bacterial RedAm. In addition, we found that the recombinant soluble expression of pIR23 or *Bac*RedAm in *Escherichia coli* (BL21) resulted in high protein yield making their production and isolation easy and straightforward. These factors encouraged us to further study pIR23’s and *Bac*RedAm’s catalytic properties for the reductive amination of α,β-unsaturated carbonyl compounds.

### Kinetic Parameters for pIR23 and BacRedAm-Catalyzed Reductive Amination of (Hydro)cinnamaldehyde

Using *Bac*RedAm as a prototype bacterial reductive aminase, we determined and compared kinetic parameters of pIR23 *v*s *Bac*RedAm for model RedAm substrate combinations, namely, cyclohexanone **1** with alkylamines (**a–e**). Both pIR23 and *Bac*RedAm displayed high NADPH-dependent reductive amination rates for cyclohexanone **1** and cyclopropylamine **a**, with *k*_cat_*of* 3.5 and 5.1 s^–1^, respectively. Similar to the reactivity pattern observed for fungal RedAms,^46^*Bac*RedAm showed comparable catalytic efficiency toward reductive amination of **1** with other alkylamines (*e.g*., propargylamine **b**, allylamine **c**, and methylamine **e**). However, pIR23 displayed significantly lower rates toward amination of cyclohexanone **1** with the alkylamines **b**, **c**, and **e** (Table 1), highlighting differences in the reactivity pattern for these substrates with different IREDs. *Bac*RedAm showed higher reactivity toward a broad range of alkylamines for the reductive amination of cyclohexanone when compared to pIR23.

**Table 1:**
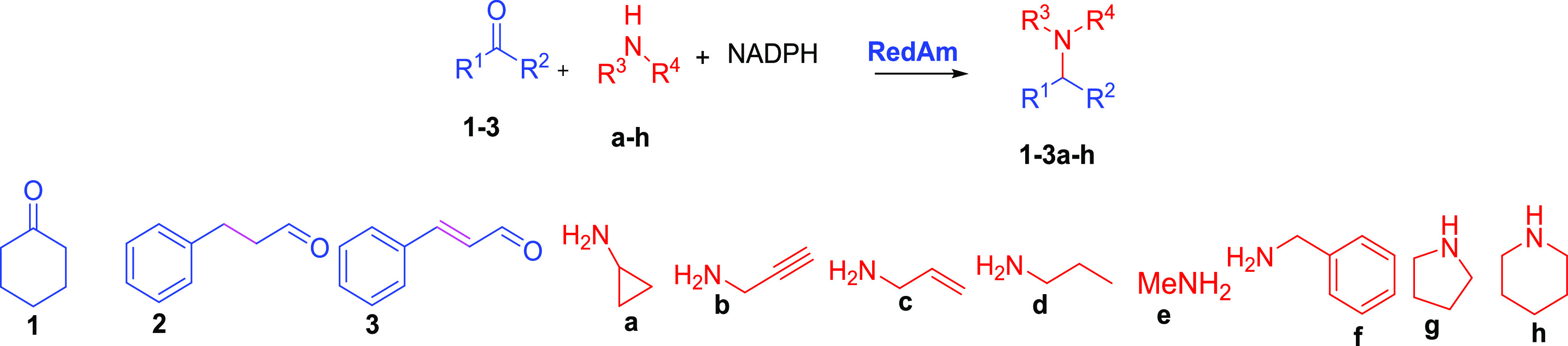
Kinetic Parameters of pIR23 and BacRedAm for Cyclohexanone, Hydrocinnamaldehyde, and Cinnamaldehyde with Different Amines^a^

			pIR23		*Bac*RedAm	
varied substrate	saturated substrates		*K*_m_ (mM)	*k*_cat_ (s^–1^)	*K*_m_ (mM)	*k*_cat_ (s^–1^)
cyclohexanone (1)	NADPH	cyclopropylamine (**a**)	10.12	3.47	13.32	5.12
		propargylamine (**b**)		0.31		3.75
		allylamine (**c**)		0.22		2.02
		methylamine (**e**)	12.35	0.94	11.41	4.74

			pIR23		*Bac*RedAm	
varied substrate	saturated substrates		*K*_m_ (mM)	*k*_cat_ (s^–1^)	*K*_m_ (mM)	*k*_cat_ (s^–1^)
hydrocinnamaldehyde (2)	NADPH	cyclopropylamine (**a**)	0.43	1.57	2.98	1.45
		propargylamine (**b**)	0.10	0.89	2.43	1.95
		allylamine (**c**)	0.33	1.00	3.32	2.01
		propylamine (**d**)	0.64	1.26	n.d.	n.d.
		methylamine (**e**)	0.32	0.46	2.12	0.61
		benzylamine (**f**)	0.89	0.81	2.14	0.10
		pyrrolidine (**g**)	0.64	1.84	2.54	0.13
		piperidine (**h**)	0.89	0.64	n.d.	n.d.

			pIR23		*Bac*RedAm	
varied substrate	saturated substrates		*K*_m_ (mM)	*k*_cat_ (s^–1^)	*K*_m_ (mM)	*k*_cat_ (s^–1^)
cinnamaldehyde (3)	NADPH	cyclopropylamine (**a**)	0.20	0.36	2.58	0.11
		propargylamine (**b**)	0.12	0.23	1.96	0.09
		allylamine (**c**)	0.33	0.27	1.89	0.10
		propylamine (**d**)	0.77	0.48	n.d.	n.d.
		methylamine (**e**)	0.27	0.13	2.54	0.04
		benzylamine (**f**)	0.29	0.15	2.45	0.08
		pyrrolidine (**g**)	0.25	0.77	3.34	0.20
		piperidine (**h**)	0.34	0.25	n.d.	n.d.

a One unit of activity = the amount of pure enzyme required to consume 1 μmol of NADPH per min. The initial rate activity measurements were performed at pH 7.5 (100 mM NaP*i* buffer), Temp = 30 °C; [NADPH] = 0.3 mM; [amine] = 80 mM. n.d.—not determined. Activity measurements were performed in triplicate; Table S2 (Supporting Information) provides data with error/standard deviation from the mean activity (*k*_cat_) or mean *K*_m_ values.

We next examined the catalytic performance of pIR23 and *Bac*RedAm toward the reductive amination of a prototype α,β-unsaturated aldehyde, cinnamaldehyde **3**. Steady-state kinetic measurements were performed at various concentrations of cinnamaldehyde **3**, while cyclopropylamine **a** and NADPH were maintained at saturating concentrations, 80 and 0.3 mM, respectively. Similarly, kinetic parameters were also determined with other primary amines including propargylamine **b**, allylamine **c**, propylamine **d**, methylamine **e**, benzylamine **f** as well as secondary amines, pyrrolidine **g**, and piperidine **h**. To allow us to gauge the effect of conjugation on the catalytic rates, we also determined the kinetic parameters for the saturated analogue, hydrocinnamaldehyde **2**, again with amines **a–h**.

In general, pIR23 exhibited modest reductive amination rates toward cinnamaldehyde with different amine nucleophiles reaching up to a *k*_cat_ of 0.77 s^–1^. In comparison, amination rates for hydrocinnamaldehyde proceeded about 3–5 times faster under the same conditions, with a highest activity of 1.84 s^–1^ achieved (Table 1). The binding affinities for the two carbonyl acceptors were comparable as deduced from the similar *K*_m_ values. This suggests that the differences in the amination efficiency for the analogous aldehydes are not a result of the differing binding behavior of these two related substrates, rather a possible consequence of the stabilizing effect of the adjacent C=C in cinnamaldehyde. The reactivity of amines **a**–**h** followed a similar trend for both aldehydes; *N*-alkyl primary amines such as **a**–**d** were readily tolerated by pIR23, whereas methylamine **d** and relatively bulky amines (*e.g*., benzylamine, **f**) were less reactive. Similarly, pIR23 showed tolerance for five- and six-membered cyclic secondary amines, pyrrolidine **g** and piperidine **h**, displaying a clear preference for **g**.

On the other hand, *Bac*RedAm displays a comparable catalytic rate to pIR23 for the amination of the hydrocinnamaldehyde **2** but exhibits weak activity (up to an order of a magnitude lower in catalytic velocity) toward the amination of the cinnamaldehyde **3** (Table 1). This clearly establishes pIR23 as the better catalyst for the reductive amination of α,β-unsaturated carbonyl compounds, so further studies for this substrate group were performed with pIR23.

### pIR23-Catalyzed Reductive Amination Provides Access to (Allylic) Secondary and Tertiary Amines

**Figure 2:**
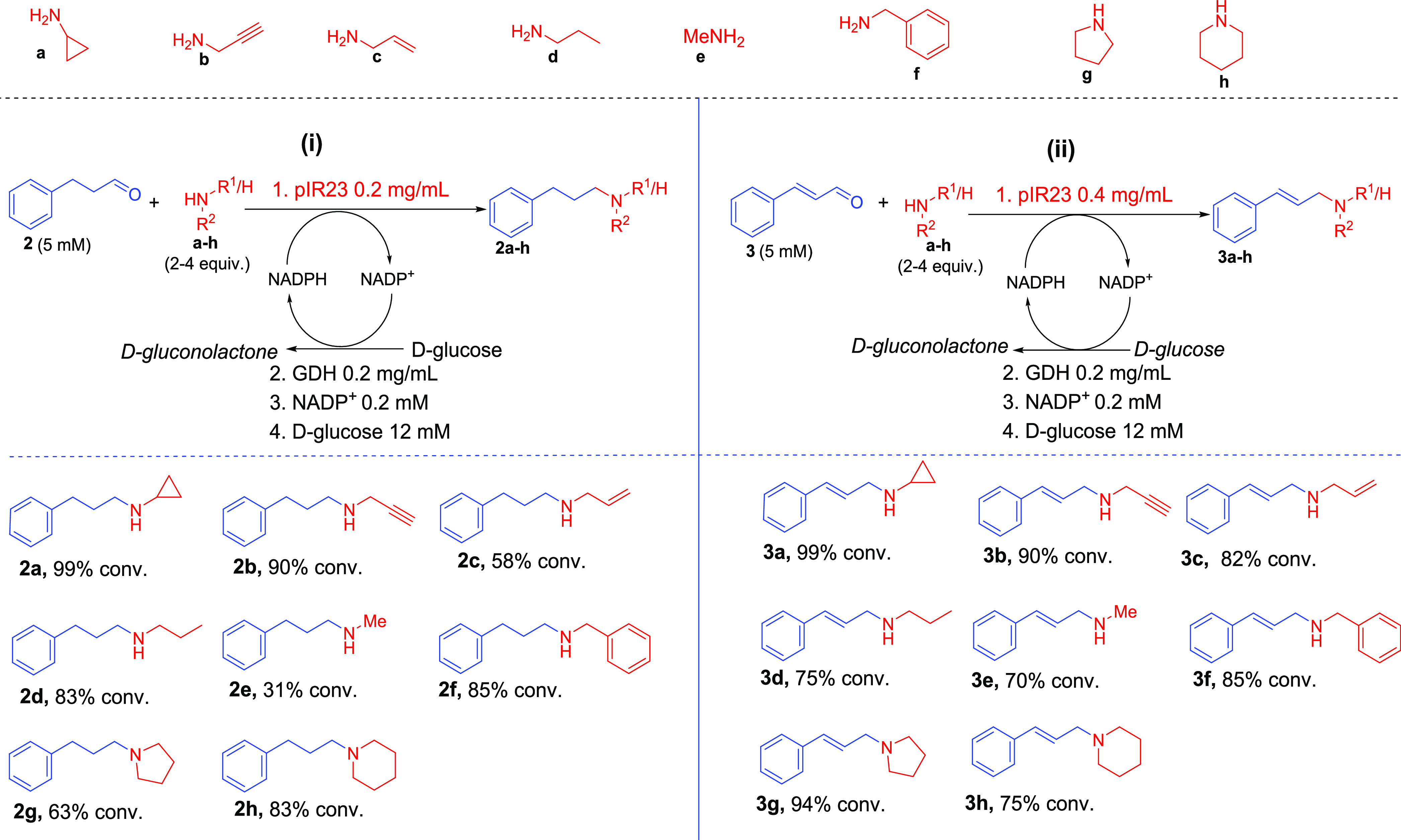
Biocatalytic reductive amination for the preparation of secondary and tertiary allylic amines and their saturated analogues. Reaction conditions as specified in the scheme. Amines a, b, c, d, and g were supplied as 2 equiv, whereas amines e and h were supplied as 4 equiv. Reactions were run in 50 mM NaP*i* buffer, pH 7.5, incubated for 6 h at 30 °C, 250 rpm shaking. Only 0.5–8% alcohol product was detected. Enzymes: pIR23 = *C. ferrugineus* reductive aminase; GDH = *Bacillus subtilis* glucose dehydrogenase.

To investigate the synthetic applicability of this enzyme further, a series of biotransformation reactions were performed for the amination of hydrocinnamaldehyde with amines **a**–**h** as coupling partners, monitoring product formation using GC-MS. A glucose dehydrogenase (GDH)-based NADPH-recycling system was coupled to the pIR23-catalyzed reaction, enabling regeneration and supply of NADPH. Through this system, hydrocinnamaldehyde (1 equiv) was coupled to various amines **a–h** (2–4 equiv), furnishing the corresponding *N*-alkylated secondary and tertiary 3-phenylpropylamine derivatives (**2a**–**1h)** in up to 99% conversion (Figure 2).

We next examined reductive amination of cinnamaldehyde **3** again with amines **a**–**h** to generate the allylic amine analogues. The desired allylic amines **3a**–**2h** were obtained in conversions between 70 and 99% (Figure 2). Analysis of enzyme-free controls featuring all other reaction conditions lacked the amine product peaks. Instead, depending on the amine coupling partner, the conjugated imine condensation products could be detected (Table 2).

**Table 2:**
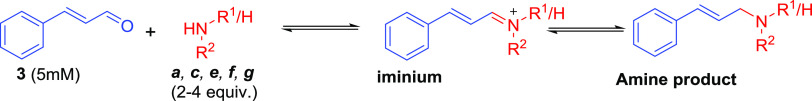

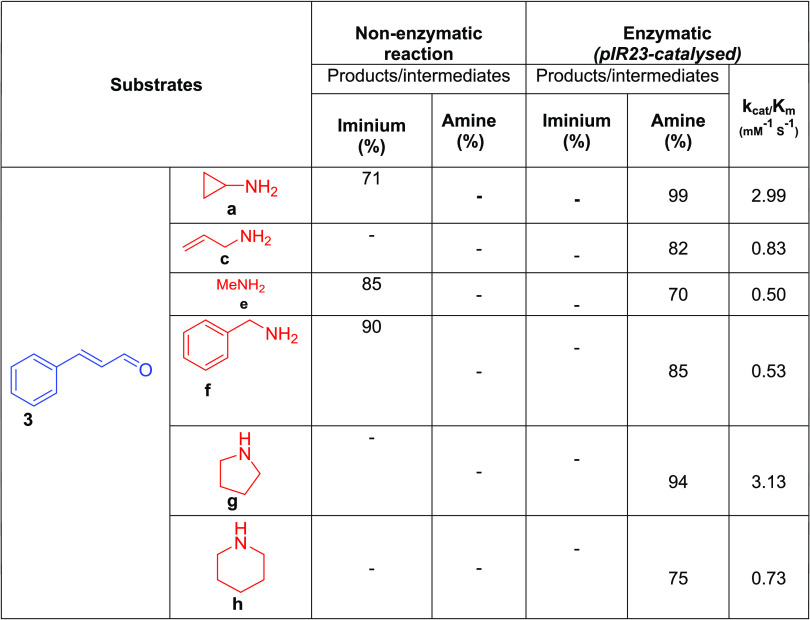
Analysis of Efficiency of pIR23-Catalyzed Reductive Amination for Substrates Where Spontaneous Imine Formation Was Quantitatively Observed *vs* Substrate Combinations Where Spontaneously Formed Imine Intermediates Were Undetectable^a^

a **[-]** = not detected. 2 equiv amines **a, c, f**, and **g** and 4 equiv amines **e** and **h** were supplied for both enzymatic and nonenzymatic reactions. Reactions were run in 50 mM NaP*i* buffer, pH 7.5. All reactions were incubated for 6 h at 30 °C, 250 rpm shaking. Enzyme reaction contained *C. ferrugineus* reductive aminase (pIR23) 0.4mg mL^–1^; *B. subtilis* glucose dehydrogenase (*Bs*GDH) 0.2 mg mL^–1^, NADP^+^. Only 0.5–15% alcohol product was detected.

### pIR23 Is Self-Sufficient in Catalyzing Formal Reductive Amination

Formal reductive amination is a two-step process comprising initial imine formation (from the corresponding carbonyl compound and amine) and a subsequent C=N reduction step. Although the initial step can occur spontaneously, in water formation of exocyclic imines is hampered by the thermodynamically favored imine hydrolysis, reverting the intermediate imine to its starting components. As such, it has been difficult to monitor exocyclic imines in aqueous enzymatic reaction systems.^53,54^ We have previously shown that reductive aminases, a subset of fungal IREDs, can accelerate the imine formation step as well as efficiently reduce the formed imine, making efficient equimolar reductive amination feasible,^46,47^ even at an industrial scale.^48,55,56^ Recently, out of the numerous bacterial IREDs characterized, few have been shown to behave as reductive aminases,^48−50^ catalyzing reductive amination of some carbonyl compounds with equimolar amounts of amines. However, evidence to pinpoint their ability to catalyze imine formation is lacking.

In the light of this, we aimed to examine whether the bacterial pIR23 relies principally on the nonenzymatic spontaneous imine formation in the reductive amination process. We anticipated that the conjugation in the imine intermediates generated by coupling of cinnamaldehyde with the amines **a**–**h** is likely to confer more stability, making them less prone to hydrolysis in the aqueous reaction buffer. Indeed, GC-MS analysis of enzyme-free reactions following 6 h incubation of cinnamaldehyde with the amines **a**, **e**, and **f** in reaction buffer, under the same biotransformation conditions, quantitively formed the corresponding imines in 71, 85, and 90% conversion (relative to the cinnamaldehyde), respectively (Table 2). However, no imine intermediate was detected for the enzyme-free reaction of cinnamaldehyde with **c, g**, or **h**. In all cases, the corresponding amine product peak was not detected in these enzyme-free reactions (Table 2).

A comparison of catalytic efficiency and reductive amination conversion values for pIR23-catalyzed amination *vs* the amount of spontaneously (nonenzymatic) formed imine for related substrates (Table 2) clearly showed that pIR23-catalyzed amination efficiency is not hampered by the rate of nonenzymatic imine formation/hydrolysis. Conversion values were comparable for reactions for which a significant amount of the imine intermediate can be spontaneously formed (*e.g*., cinnamaldehyde with **a** or cinnamaldehyde with **e**) with those for which spontaneous imine formation is less favored (cinnamaldehyde +**c**, cinnamaldehyde +**g**, cinnamaldehyde +**h**). This indicates that pIR23 is self-sufficient in catalyzing a formal reductive amination and represents a prototype bacterial reductive aminase.

**Figure 3:**
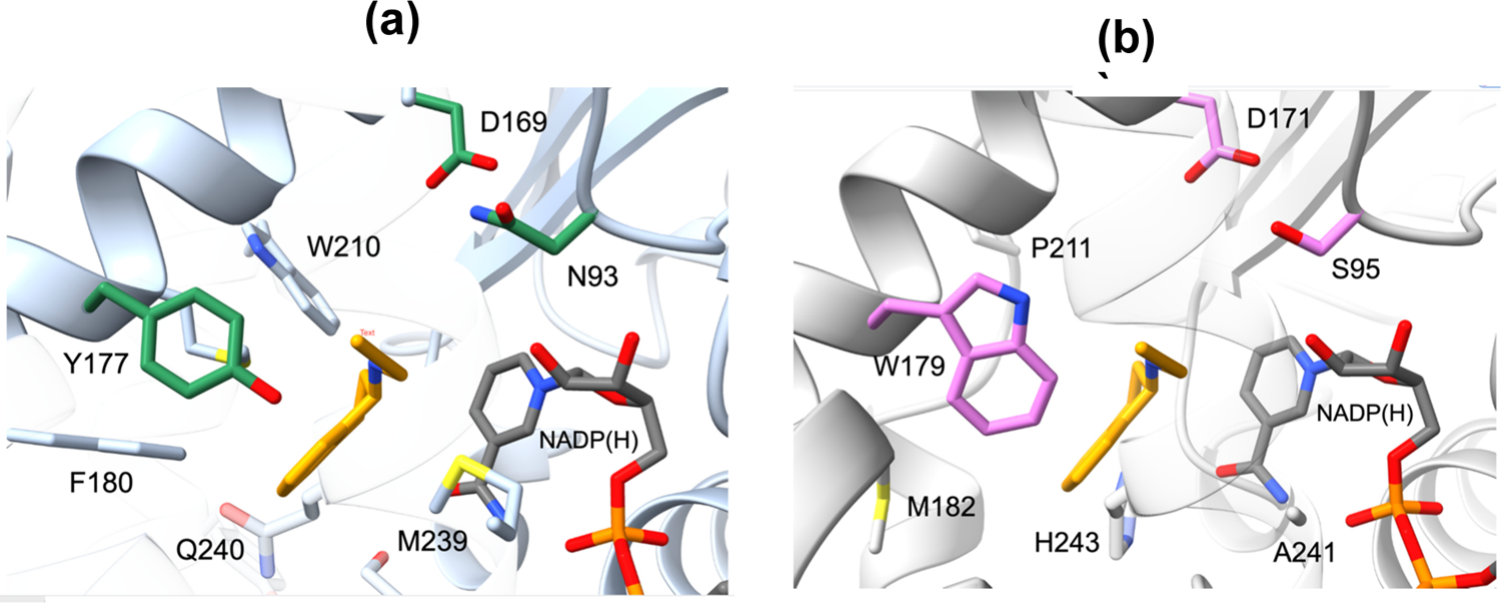
Comparison of structures of *Asp*RedAm in complex with NADP(H) and rasagaline (a)^46^ and a homology model of pIR23 (b) created using SWISS-MODEL^58^ and visualized with ChimeraX.^59^ Essential catalytic residues in *Asp*RedAm include N93, D169, Y177, which are also conserved in closely related fungal homologues. Equivalent positions in pIR23 feature S95 (S, N, or T in other bacterial homologues), D171, and W179 (W, F, or Y in other bacterial homologues), respectively (Supporting Information Table S3).

So far insights into RedAm catalysis have largely come from mechanistic studies of fungal reductive aminases.^46,47^ In our previous studies on the reductive aminase from*Aspergillus oryzae* (*Asp*RedAm) and related fungal homologues, we established that these enzymes mediate reductive amination by employing three essential active site residues, namely, D169, Y177, and N93 (Figure 3), which are conserved in fungal RedAms.^46,47^ While Y177 coordinates the carbonyl group of the ketone, D169 deprotonates the amine, facilitating a nucleophilic attack *en route* the key carbinolamine intermediate (see ref (47) for the proposed RedAm catalytic mechanism).^47^ Interestingly, these residues are conserved in the prototype bacterial reductive aminase *Bac*RedAm and can explain why this enzyme exhibits a similar reactivity pattern and amine substrate scope with fungal RedAms despite being only being ∼50% identical to the fungal RedAms by primary sequence comparison. However, in most other bacterial IREDs catalyzing equimolar reductive amination, only D169 is conserved (D171 in pIR23), while N93 is often replaced by other polar residues of comparable size such as serine or threonine (S95 in pIR23) (Supporting Information Table S3), and Y177 position (*Asp*RedAm numbering) often features tryptophan or phenylalanine in many bacterial IREDs including pIR23 (W179) (Figure 3, Supporting Information Table S3). Given that the phenolic moiety of Y177 and its conjugate base, the phenolate anion has been proposed to be involved in the fungal RedAm-mediated reductive amination process, it is unclear if these roles are performed by other active site residues. In this case, the significant dynamic changes and domain flexibility which are well-established features of IREDs/RedAms’ catalysis^46,47,57^ should facilitate the substrate/intermediate (re)positioning. Alternatively, it is possible that the proposed roles of Y177 are performed by a water molecule, and hence, the electronic property of residues at 177 (equivalent) position is of little significance in this catalysis. The interesting catalytic features of pIR23 and other emerging bacterial reductive aminases warrant a detailed study of their catalytic mechanism.

### *N*-Allylation of Primary and Secondary Amines with Acrylic Acids Using pIR23

**Figure 4:**
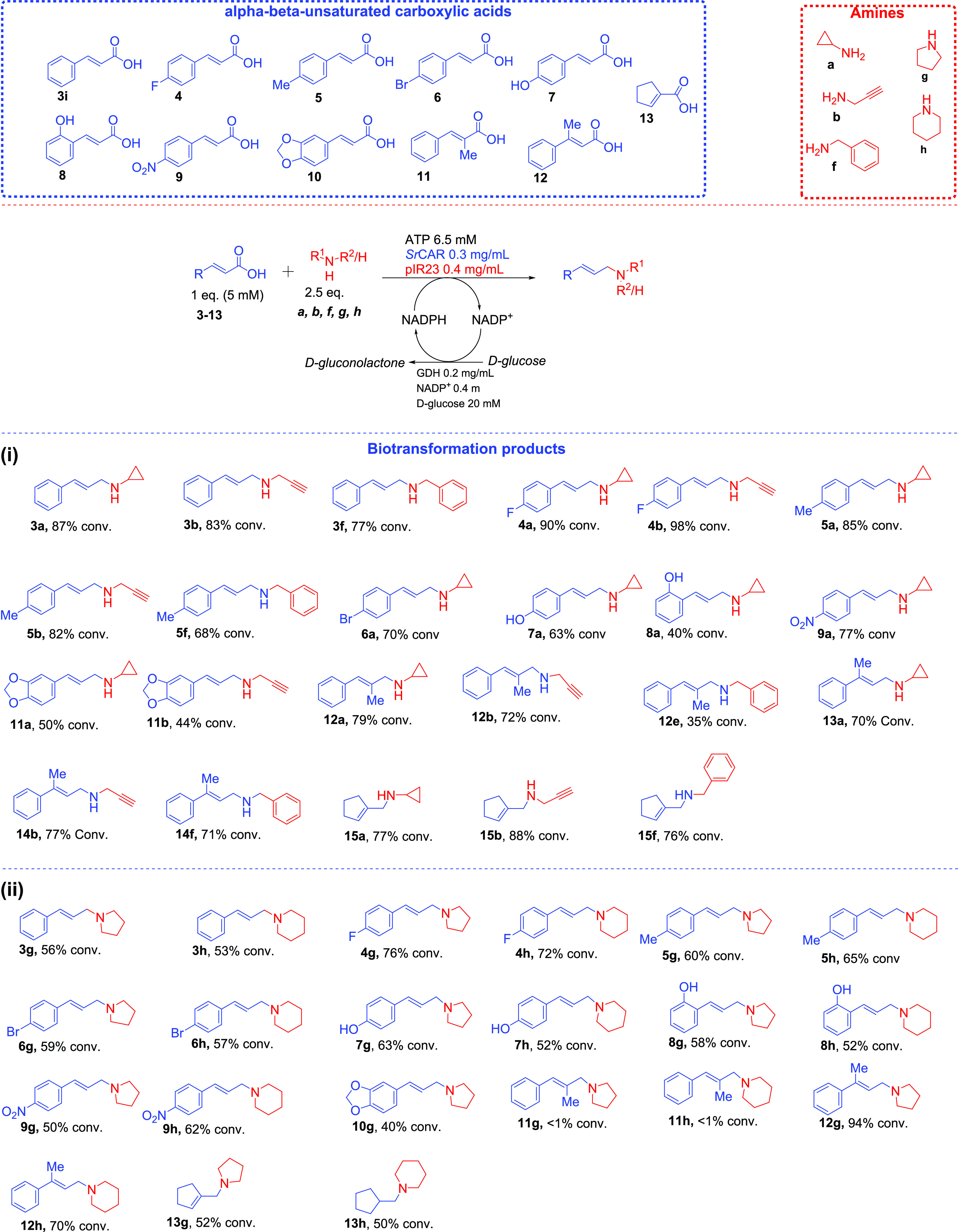
One-pot biocatalytic system for the *N*-allylation of primary amines and secondary amines with acrylic acid derivatives, generating the corresponding (i) allylic secondary amines and (ii) allylic tertiary amines, respectively. The one-pot two-step system involves an *in situSr*CAR-catalyzed carboxylate reduction yielding an α,β-unsaturated aldehyde, which is then coupled with a primary or secondary amine in the same pot via pIR23-catalyzed reductive amination. Reaction conditions have been specified in the scheme. Carboxylic acid was used at 5 mM (1 equiv), while amine was supplied at 2.5 equiv. Reactions were performed in NaP*i* buffer (50 mM, pH 7.5, supplemented with 10 mM MgCl_2_ and 2% v/v DMSO). Reactions were incubated at 30 °C, 250 rpm for 18 h. For substrates **6**, **9**, and **10**, 1–8% corresponding saturated amine products were detected. Typically, 0.5–15% alcohol product detected. Conversion values were determined from GC-MS analyses. Enzymes: *SrCAR* = *S. rugosus* carboxylic acid reductase; GDH = *B. subtilis* glucose dehydrogenase; and pIR23 = *C. ferrugineus* reductive aminase. All enzymes were used as purified preparation.

Satisfied with the conversion values obtained in reductive amination of cinnamaldehyde, we next examined direct coupling of cinnamic acid **3i** with the primary amine **a** in a one-pot process *via* the aldehyde intermediate generated *in situ* from the broad-scope *Segniliparus rugosus* carboxylic acid reductase (*Sr*CAR).^41,45^ Again, a GDH-based NADPH-recycling system was incorporated to regenerate NADPH from glucose (as NADPH is required in both the *Sr*CAR and pIR23-mediated steps), while a stoichiometric amount of ATP was supplied. Using this system, cinnamic acid **3i** (1 equiv) was aminated with an amine **a** (2 equiv) to generate the corresponding secondary amine **3a** in 87% conversion. To investigate the scope of the CAR-RedAm system, direct amination of a variety of acrylic acid derivatives **3i, 4–13** with three primary amines **a**, **b**, and **f** was undertaken. The resulting secondary allylic amine products were formed from the coupling of acids **3i, 4–13** with **a**, **b**, and **f**, affording moderate to excellent conversions of up 98% (Figure 4).

Cinnamic acid **3i** and derivatives bearing *p*-substituents on the aromatic ring such as weakly electron-withdrawing halogens (*p*-F, **4**, *p*-Br, **6**), weakly electron-donating groups such as Me (**5**), as well as strong electron-donating group (*e.g., p*-OH, **7**; *o*-OH, **8**) or strong electron-withdrawing groups (*e.g*., *p*-NO_2_, **9**), were accepted. The reactions afforded the allylic amine products in moderate to excellent conversions (25–98% conversion), depending on the carboxylate/amine coupling partners. Difunctionalized cinnamic acid derivative **10** was coupled to the amines **a** and **b**, furnishing the corresponding amines in 50 and 59% conversions, respectively. Furthermore, cinnamic acid derivatives bearing small substituents at the α- or β-carbons to the carboxylate (α-Me **11, β-**Me **12**) were also accepted in up to 79% conversion. Similarly, α,β-unsaturated cyclic carboxylic acids **13** generated the corresponding allylic amines in high conversions (76–88%).

In line with kinetic data from Table 1, amines **a** and **b** performed as better nucleophiles than the bulky amine **f**. Hence, **a** and **b** were readily alkylated, as evident by the high conversion values (Figure 4i). Carboxylic acid substrates, which were previously shown to exhibit high reactivity with *Sr*CAR (*e.g*., **3i, 4, 5**, **11**–**13**),^41^ were excellent alkyl donors, provided the amine nucleophile was highly reactive in the pIR23-catalyzed step (Figure 4i). We observed that in instances where a highly reactive alkyl source (excellent CAR substrate) was incubated with a poorly reactive amine (*e.g*., benzylamine **f**), the aldehyde intermediate accumulated and was often converted to the corresponding primary alcohol upon long incubation (due to the weak promiscuous carbonyl reductase activity of GDH as has previously been observed).^41^ Similarly, lower conversion values were observed when excellent amine nucleophiles (*e.g*., **a** and **b**) were paired with poorly reactive carboxylic acid substrates of CAR (poor alkylating agents), as significant residual carboxylate starting material was detected in these biotransformations. Conversely, by combining a good CAR substrate (as alkylating substrate) with a highly reactive amine nucleophile, the *N*-allylation reaction proceeds with high efficiency, forming the allylic products at >70% conversion (Figure 4i). Although a stoichiometric amount of ATP has been used in the analytical one-pot *Sr*CAR-*pIR23* system, a straightforward ATP cofactor regeneration system based on a family-2 polyphosphate kinase (PPK2)^43,60^ has been employed to recycle ATP in the preparative biotransformation reactions.

Encouraged by the high conversion obtained toward the synthesis of allylic secondary amines, we next aimed to extend our system to the synthesis of tertiary allylic amines. To our knowledge, direct enzymatic amination of carboxylic acids to generate (allylic) tertiary amines has yet to be demonstrated. In view of the prevalence of (allylic) tertiary amine moieties in bioactive compounds, especially those containing pyrrolidine and piperidine frameworks, we envisaged that a selective and sustainable catalytic method for the conversion of biomass-derived carboxylates to allylic tertiary amine scaffolds would be synthetically attractive. Hence, we applied the CAR-pIR23 system for the direct reductive *N*-allylation of secondary amines using pyrrolidine **g** and piperidine **h** with acrylic acids **3i, 4–13** (Figure 4ii). Impressively, a wide range of cinnamic acid derivatives were coupled with pyrrolidine and piperidine to generate the corresponding allylic pyrrolidine derivatives (**3g**–**13g**, 40–94% conversion) and allylic piperidine derivatives **(3h**–**13h**, 50–76% conversion) (Figure 4ii). The reactivity pattern follows the trend observed in Figure 4i, except for α-Me cinnamic acid **11**, where the corresponding aldehyde intermediate did not couple with either **g** or **h**, due to a possible steric hindrance from the α-substituent. In contrast, β-Me cinnamic acid **12** was an excellent alkyl source for both amines **g** and **h**, generating the corresponding allylic **12g** and **12h** in 94 and 70% conversion, respectively. Similarly, α,β-unsaturated cyclic carboxylic acid **13** was successfully coupled with both **g** and **h** to yield the corresponding allylic tertiary amines, **13g** and **13h** in ≥50% conversion (Figure 4ii).

### Preparative Scale Reactions

We sought to demonstrate the preparative applicability of pIR23-catalyzed reductive amination of α,β-unsaturated aldehydes, as well as the one-pot biocatalytic *N*-allylation using carboxylic acids as allylating agents. We observed limited solubility of cinnamaldehyde in aqueous buffer at ≥20 mM (despite the addition of 2–4% DMSO as a cosolvent), so pIR23-catalyzed reductive amination of cinnamaldehyde (100 mg scale) was performed at 15 mM substrate loading. A preparative (100 mg scale) reductive amination of **3** with 2.5 equiv of amines **a**, **b**, and **g** catalyzed by pIR23, coupled to a GDH-mediated NADPH recycling, was successfully performed, yielding *N*-cinnamylcyclopropanamine (**3a**, 68% isolated yield), *N*-cinnamylpropargylamine (**3b**, 70% isolated yield), and *N*-cinnamylpyrrolidine (**3g**, 61% isolated yield), respectively, after 18 h (Table 3). Further intensification of this reaction may be achieved if a more suitable cosolvent is identified, from a comprehensive screening of green nonaqueous cosolvents. Such screening may reveal compatible cosolvents that readily dissolve the substrate(s) and are noninhibitory to the biocatalysts employed in the cascade, for example, deep-eutectic solvents^61,62^ or cyclopentyl methyl ether.^63^

**Table 3:**
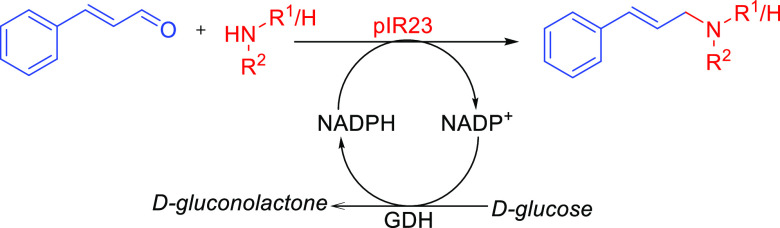

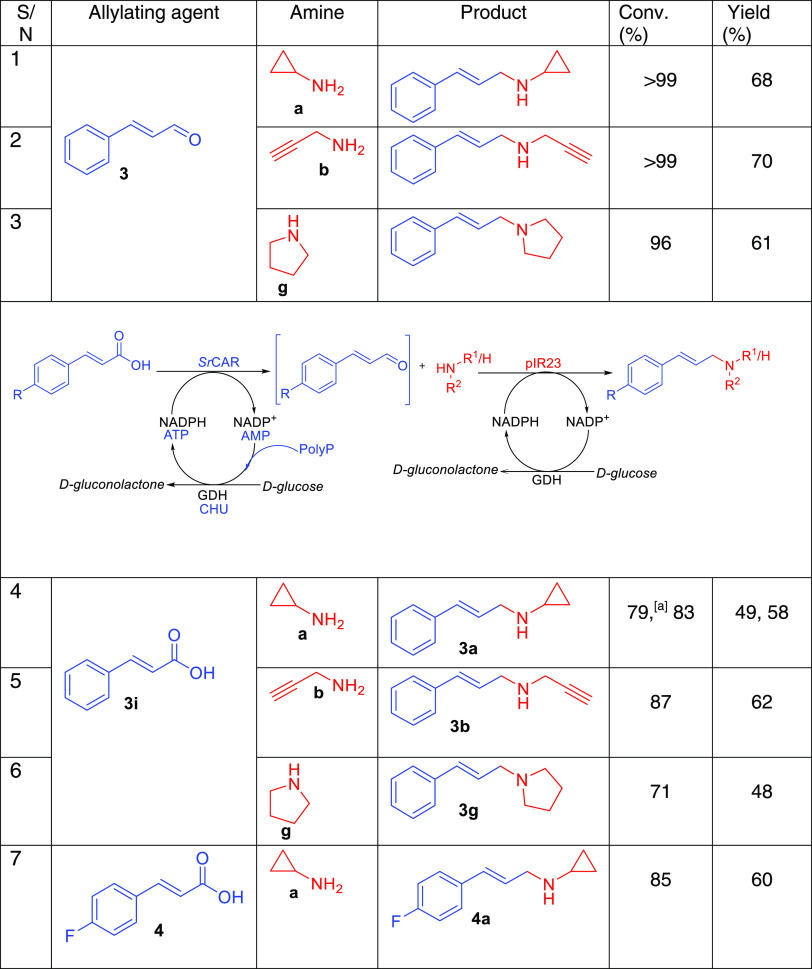
Examples of Preparative Biotransformation Reactions for RedAm-Catalyzed Reductive Amination of Cinnamaldehyde and CAR-RedAm One-Pot Allylation of Amines with Cinnamic Acids Providing Access to 2°and 3° Allylic Amines^a^

a All preparative reactions (100 mg of aldehyde or carboxylic acid) were performed with catalytic amounts of cofactors (0.5 mM NADP^+^ and 0.2 mM ATP) using respective cofactor recycling system(s) except [a], which was performed with a stoichiometric supply of ATP (2 equiv).

For the *Sr*CAR-pIR23-mediated one-pot *N*-allylation of **a** with cinnamic acid **3i**, we initially performed the preparative reaction using conditions established for analytical biotransformation with stoichiometric amount of ATP (2 equiv). Under these conditions, the one-pot *Sr*CAR-pIR23 biotransformation reaction containing 100 mg of cinnamic acid and 2.5 equiv **a** as starting materials yielded *N*-cinnamylcyclopropanamine **3a** (79 conv., 49% isolated yield following 21 h incubation). We next replaced stoichiometric ATP with a CHU kinase-mediated ATP-recycling system. CHU is a member of the PPK2 family and catalyzes the phosphorylation of AMP to ATP (*via* ADP intermediate) using the inexpensive polyphosphate (polyP) as the phosphate donor.^60^ Thus, the preparative one-pot *Sr*CAR-pIR23 amination of cinnamic acid (100 mg) with cyclopropylamine **a** (2.5 equiv) coupled to GDH-based NADPH recycling and CHU-mediated ATP-recycling afforded *N*-cinnamylcyclopropanamine **3a** (58% isolated yield). This system also allowed the coupling of cinnamic acid with propargylamine **b** and pyrrolidine **g**, yielding the corresponding allylic amines *N*-cinnamylpropargylamine (**3b**, 62% isolated yield) and *N*-cinnamylpyrrolidine (**3g**, 48% isolated yield), respectively. Using similar reaction conditions, *p*-F cinnamic acid **4** was aminated with **a** to yield the corresponding allylic amine **4a** (60% isolated yield) (Table 3).

In recent times, efforts have been intensified toward the valorization of biomass derivatives as a strategy to promote sustainable chemical synthesis.^64^ Conversion of biomass-derived starting materials to amines has only recently received attention,^61,65−67^and bioconversion methods have largely focused on the amination of biomass-derived aldehydes/ketones to generate primary amines.^61,67^ Secondary and tertiary amines are frequently encountered as structural components in numerous bioactive compounds (*e.g*., pharmaceuticals, agrochemicals, and natural products). Therefore, access to selective catalytic strategies enabling the conversion of renewable starting materials to industrially useful secondary and tertiary (allylic) amines under environmentally friendly conditions would facilitate the transition to sustainable, resource-efficient manufacturing of these amine-based building blocks. Such methods can have a tremendous impact on sustainable synthesis since secondary and tertiary (allylic) amine frameworks constitute a significant proportion of the amine chemical space.

Our *N*-allylation method contributes to the sustainable synthesis of allylic amines in two respects. By demonstrating direct access to secondary and tertiary allylic amines under mild enzymatic-catalyzed transformations, we extend the synthetic scope of enzymatic allylic amine syntheses beyond allylic primary amines. Our method provides an alternative green synthesis route to secondary and tertiary allylic amines through biocatalysis, a powerful synthesis method, which is widely considered green and sustainable.^68^ The other merit is the use of renewable starting reagents. Our choice of biomass-derivable renewable cinnamic acids as alkylating agents highlights alternative green reagents for *N*-allylation of alkylamines.

Cinnamic acid and derivatives (*e.g*., *p*-coumaric acid, ferulic acid, caffeic acid) can sustainably be sourced from biomass. For example, several microbial systems have been developed allowing economically viable fermentative production of cinnamic acids from l-phenylalanine/tyrosine.^69,70^ Although access to non-natural cinnamic acid derivatives from biomass have been less explored, recent work has shown that non-natural cinnamic acid derivatives can be accessed from microbial cultures fed with unnatural amino acid precursors (e.g., halogenated phenylalanines or tyrosines),^71^ pointing to future prospect of microbial *de novo* production of non-natural substituted cinnamic acids. These developments will expand the skeletal diversity of renewable allylating reagents, potentially reducing overdependence on petrochemicals for these reagents. The availability of green, straightforward catalytic methods as demonstrated in this work should enable a completely sustainable process for the production of structurally diverse allylic amines from simple starting (non)-natural renewable starting materials.

## Conclusions

In summary, we have provided evidence that pIR23, a prototype bacterial reductive aminase, is self-sufficient in catalyzing a formal reductive amination of α,β-unsaturated aldehydes with various amines, generating a broad range of secondary and tertiary amines. Another novel prototype bacterial reductive aminase *Bac*RedAm bearing identical active site residues to those conserved in fungal RedAms was shown to exhibit a similar reactivity pattern to these RedAms. BacRedAm displayed a high catalytic reductive amination rates for cyclohexanone and hydrocinnamaldehyde but interestingly exhibited only weak activity toward cinnamaldehyde; the latter was efficiently aminated by the pIR23 enzyme. This highlights a clear difference in the reactivity of different RedAms toward the amination of α,β-unsaturated carbonyl compounds *vs* their saturated analogues. By applying pIR23, a robust system for the direct reductive *N*-allylation of primary and secondary amines using readily accessible biomass-derivable acrylic acids, has been developed. The two-step one-pot system comprises an initial carboxylate reduction to generate the corresponding α,β-unsaturated aldehyde *in situ*, followed by reductive amination of the unsaturated aldehyde to yield the corresponding allylic amine. Using this approach, several secondary and tertiary allylic amines were accessed in high conversions from a broad range of acrylic acids, including seven selected preparative scale examples. This process represents a green and selective approach (avoiding overalkylation and over reduction of the adjacent alkene bond), using renewable carboxylic acids for the synthesis of a wide range of secondary and tertiary allylic amine frameworks under mild reaction conditions. We envisage that this system can inspire future cascades for the preparation of allylic amines from simple starting materials. For example, coupling the CAR-RedAm system to an upstream biosynthetic pathway for the *de novo* synthesis of cinnamic acids^72^ or to a CO_2_-fixing enzyme.^73,74^ Although the present study only focused on the synthesis of linear secondary and tertiary allylic amines, our future interests include identifying and developing suitable biocatalysts for the asymmetric version of this transformation using ultra-high-throughput enzyme screening technologies.^75^

## Supporting Information Available

Experimental procedures, protein sequences, nonlinear regression analysis plots from enzyme kinetic data, sample chromatograms, and NMR spectra of biotransformation reactions (PDF)
