# Supplementary material for: Enzymatic N-Allylation of Primary and Secondary Amines Using Renewable Cinnamic Acids Enabled by Bacterial Reductive Aminases
Source: ACS Sustain Chem Eng. 2022 May 6;10(20):6794–806. doi: 10.1021/acssuschemeng.2c01180 (PMC9131517; doi:10.1021/acssuschemeng.2c01180)
Supplement: Supplementary file 1 — sc2c01180_si_001.pdf [file sc2c01180_si_001.pdf]

Supporting Information

Enzymatic N-allylation of primary and secondary amines using renewable cinnamic acids enabled by bacterial reductive aminases

Godwin A. Aleku,[†, ‡]* Gabriel R. Titchiner,[†] George W. Roberts,[†] Sasha R. Derrington,[†] James R. Marshall,[†] Florian Hollfelder,[‡] Nicholas J. Turner,[†]* and David Leys[†]*

[†]*Manchester Institute of Biotechnology, Department of Chemistry, University of Manchester, Manchester, 131 Princess Street, Manchester, M1 7DN, UK.*

Email: David.Leys@manchester.ac.uk

Email: Nicholas.Turner@manchester.ac.uk

[‡]*Department of Biochemistry, University of Cambridge, 80 Tennis Court Road, Cambridge, CB2 1GA, UK.*

Email: ga430@cam.ac.uk

Table of Contents

Section S1. Experimental procedures S3

1.1 Chemicals S3

1.2 Production and purification of SrCAR S3

1.3 Production and purification of pIR23 and BacRedAm S4

1.4 Screening and identification of a suitable reductive aminases S4

1.5 Determination of kinetic parameters S8

1.6 Biotransformation for reductive amination of aldehydes S8

1.7 One-pot biotransformation for N-allylation of amines with acrylic acids S8

1.8 GC-MS analysis S9

Section S2. Protein sequence of IREDs/RedAms S9

Section S3. Regression analysis plots from kinetic activity measurement of pIR23 reductive amination activity (representative plots presented) S10

Section S4. Analyses of GC-MS chromatograms of reductive amination reactions S14

Section S5. Analyses of GC-MS chromatograms one-pot reductive amination of carboxylic acids S19

Section S6. Preparative Biotransformations S25

Section 7. NMR spectra of products isolated form CAR-RedAm one-pot cascade for the amination of cinnamic acids with alkylamines S28

References S32

List of Tables

Table S1. Kinetic parameters of BacRedAm on selected substrates S6

Table S2. Kinetic parameters of pIR23 and BacRedAm for hydrocinnamaldehyde and cinnamaldehyde with different amines. S7

Table S3. Analysis of conserved catalytic residues among IREDs/RedAms catalysing equimolar reductive amination. S24

List of Figures

Figure S1. GC analysis of biotransformation reactions with the identified five IRED hits S5

Figures S2-S9. Plots and kinetic analyses from initial rate study of NADPH-dependent pIR23-catalysed of reductive amination of cinnamaldehyde and hydrocinnamaldehyde with different alkylamines S10-S13

Figures S10-S14. GC-MS analyses of enzyme-catalysed and non-enzymatic reactions of hydrocinnamadehyde with akylamines S14-S118

FigureS S15-S18. Analyses of GC-MS chromatograms one-pot reductive amination of carboxylic acids S19-S22

Section S1. Experimental procedures

1.1 Chemicals

Commercially available chemicals and reagents of highest purity were purchased from Sigma-Aldrich (Poole, Dorset, UK), or Fluorochem (Hadfield, Derbyshire, UK) unless stated otherwise. HPLC solvents were obtained from Sigma-Aldrich (Poole, Dorset, UK) or ROMIL (Waterbeach, Cambridge, UK) and GC gases from BOC gases (Guildford, UK). Enzyme nicotinamide cofactors $NADP^+$ and NADPH were purchased from Bio Basic (Markham, Ontario, Canada), and Adenosine triphosphate (ATP) and Sodium Hexametaphosphate (PolyP) was sourced from Sigma-Aldrich (Poole, Dorset, UK). LB media was purchased from Formedium (Hunstanton, UK).

1.2 Production and purification of SrCAR

Plasmids of carboxylic acid reductases *Sr*CAR and plasmid of phosphopantetheine transferase (Sfp) from *Bacillus subtilis* were sourced from in-house plasmid collections and cloned as previously reported.[1,2] *E. coli* cells were transformed with CAR and Sfp plasmids. A single colony of recombinant *E. coli* BL21 (DE3) containing pET28-b-FL-*Sr*CAR and pCDF1b-Sfp was inoculated into 10 mL lysogeny broth (LB) (1% tryptone, 0.5% yeast extract, 1% NaCl) and incubated overnight at 37 °C in an orbital shaker with 200 rpm shaking. This starter culture was used as inoculum. A 2 L flask containing 500 mL LB, supplemented with kanamycin (30 µg mL^{-1}) and streptomycin (50 µg mL^{-1}) was inoculated with 5 mL of the starter culture. Cultivation was performed at 37 °C in an orbital shaker with 200 rpm shaking. At an optical density ($OD_{600\ nm}$) of between 0.6 and 0.8, isopropyl β-D-1-thiogalactopyranoside (IPTG) was added to a final concentration of 0.2 mM to induce protein expression. Incubation was continued at 20 °C and 200 rpm for 18 h. Cells from a 500 mL culture were harvested by centrifugation at 6000 rpm for 15 min, and washed in sodium phosphate buffer (NaP*i*, 50 mM, pH 7.5). To isolate *Sr*CAR, cells were disrupted by ultrasonication at 4 °C. The enzyme was isolated from the clarified lysate by Ni-affinity chromatography using a 5 mL His-Trap HP column (GE healthcare). Desalting and buffer exchange was achieved using a PD-10 desalting column (GE Healthcare, Uppsala, Sweden), eluted protein (in 50 mM NAP*i*, pH 7.50) was concentrated using 30 kDa cut-off Centricon filters (15 mL). Protein concentration was determined using Bradford reagents with BSA of known concentration as a reference standard. The CHU kinase[3] plasmid was sourced in-house (Turner's group) and was produced and purified using the procedure described above.

1.3 Production and purification of pIR23 and BacRedAm

pIR23 cloned into pET28a+ were sourced from Turner's group enzyme collection.[4] BacRedAm gene was codon optimised for expression in *E. coli.* The condon-optimised gene

sequence was synthesized by Twist Bioscience (South San Francisco, CA 94080, United States) and cloned into pET28a+. *E. coli* cells were transformed with pET28-RedAm. A single colony of the recombinant *E. coli* BL21 (DE3) was inoculated into 10 mL LB and incubated overnight at 37 °C in an orbital shaker with 200 rpm shaking. This starter culture was used as inoculum for a 2 L flask containing 500 mL LB, supplemented with kanamycin (30 µg mL^{-1}) was inoculated with 5 mL of the starter culture. Cultivation was performed at 37 °C in an orbital shaker with 200 rpm shaking. At an optical density ($OD_{600\ nm}$) of between 0.6 and 0.8, protein production was started with addition of IPTG to the final concentration of 0.2 mM. Incubation was continued at 20°C and 200 rpm for 18 h. Cells from a 500 mL culture were harvested by centrifugation at 6000 rpm for 15 min, and washed in sodium phosphate buffer (NaP*i*, 50 mM, pH 7.5). Cells were disrupted by ultrasonication at 4 °C. The RedAm was purified from the clarified lysate by Ni-affinity chromatography using a 5 mL His-Trap HP column (GE healthcare). Desalting and buffer exchange was achieved using a PD-10 desalting column (GE Healthcare, Uppsala, Sweden), eluted protein (in 50 mM NAP*i* buffer, pH 7.5) was concentrated using 30 kDa cut-off Centricon filters (15 mL). Concentration of isolated protein was determined using the Bradford assay using BSA of known concentration as a reference standard.

1.4 Screening and identification of a suitable reductive aminases

1.4.1 Identification of pIR23

Initial screening of potential reductive aminases was performed in the reverse direction utilizing the IREDy-go colorimetric screening assay as recently reported.[4] *N*-cinnamylcyclopropanamine was used as a representative substrate for oxidative deamination. The top five IRED candidates were screened for activity in the reductive animation of cinnamaldehyde with allylamine. Reductive animation reactions were carried out in 100mM Tris pH 8.0 at a 500 ul scale in 2ml microcentrifuge tubes. The reaction mixture contained 10 mM cinnamaldehyde, 20 mM allyl amine, 4 mg mL^{-1} lyophilized IRED enzyme, 1 mg mL^{-1} lyophilized GDH enzyme (Codexis), 25mM glucose, and 0.5mM NADPH. Reactions where incubated at 30˚C for 24h, with shaking at 990rpm. Assays were quenched by the addition of 100 ul of 5M NaOH and vigorous mixed by vortexing. Samples were extracted into 1ml of MTBE following vigorous mixing and centrifugation1(3,000 x g for 1 minute), dried over anhydrous $MgSO_4$ and the now clear organic layer was subjected to GC-FID analysis.

GC analysis was performed on an Agilent 6850 GC system (Agilent, Santa Clara, CA, USA) with a flame ionization detector (FID) and autosampler. An HP-1 column with 0.32 mm inner diameter and 0.25 µm film thickness (Agilent, Santa Clara, CA, USA) was used, with the following analysis method: Inlet temperature 300˚C, 50:1 split, constant flow, Initial oven temp 100˚C, 2min hold, 30˚C per min ramp to isothermal point of 325˚C.

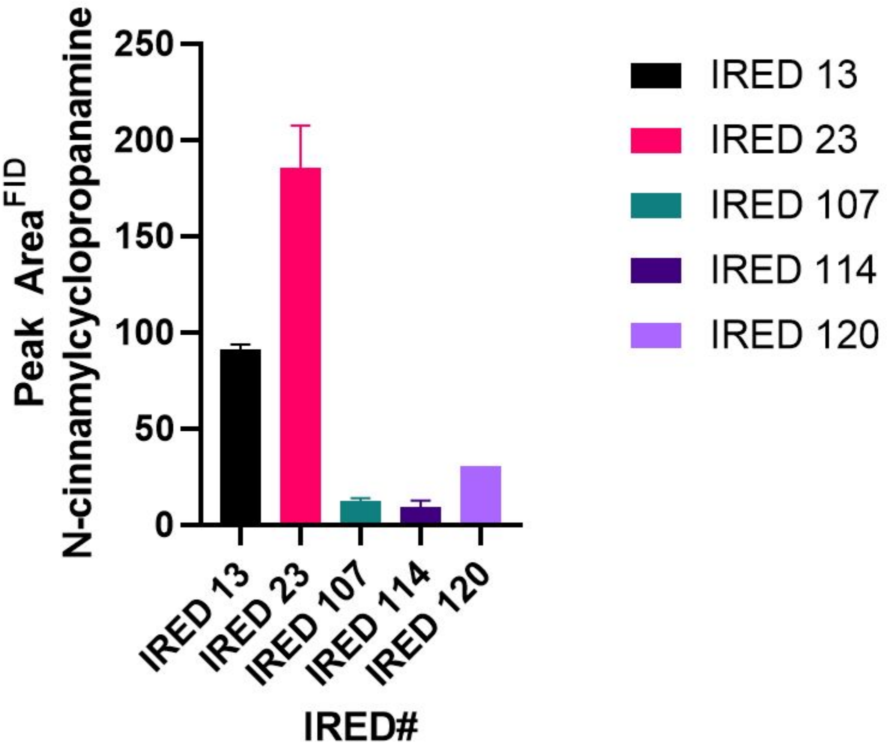

Fig. S1. Comparison of product peaks obtained after GC analysis of biotransformation reactions performed with the identified five IRED hits, revealing IR23 as best candidate.

1.4.2 Identification of BacRedAm

Using sequences of fungal reductive aminases *AspRedAm (*XP_001827659.1)[5] and *AdRedAm(*EEQ92622.1)[5], we performed blast a search against all non-reductant proteins sequences from Bacteria (taxid:2) available on the NCBI GenBank database as of September 2020. The search returned a sequence (PZN88780.1, now named BacRedAm) from a bacterium isolated from compost metagenome (Zoo Composter 4, Sao Paulo Zoo, Brazil) as the top hit (54% sequence identity, 95% query cover). The top 50 homologous bacterial sequences were further selected, and multiple sequence alignment performed using Muscle[6] showed that the relevant active site residues in fungal reductive aminases were conserved in *Bac*RedAm. Hence, BacRedAm was selected for further studies. Initial activity screening and kinetic studies reveal similar activity profile with the fungal reductive aminases (Table 1), hence BacRedAm was selected as a prototype bacterial reductive aminase.

Table S1. *Kinetic parameters of BacRedAm on selected substrates*

**Varied substrate**	**Saturated substrates**	**Km (mM)**			**kcat S^{-1}**
		NADPH	Ketone	Amine	
**NADPH**	Cyclopropylamine<br>Cyclohexanone	0.21			5.12
**Cyclohexanone**	Methylamine<br>NADPH		11.41		4.74
**4-phenylcylohexanone**	Cyclopropylamine<br>NADPH		3.22		1.56
**4-phenylbutanone**	Cyclopropylamine<br>NADPH		1.74		0.33
**Methylamine**	Cyclohexanone<br>NADPH			4.15	4.08
**Cyclopropylamine**	Cyclohexanone<br>NADPH			2.72	4.21
**Propargylamine**	Cyclohexanone<br>NADPH			2.83	3.75
**Allylamine**	Cyclohexanone<br>NADPH			9.06	2.02

Table S2. *Kinetic parameters of pIR23 and BacRedAm for hydrocinnamaldehyde and cinnamaldehyde with different amines.*

(i)

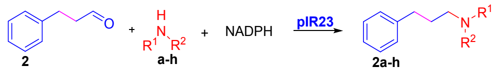

2 + a-h + NADPH → 2a-h

Varied Substrate	Saturated substrates	pIR23		*Bac*RedAm	
		k_{cat} (s^{-1})	K_m (mM)	k_{cat} (s^{-1})	K_m (mM)
Hydrocinnamaldehyde (**2**)	Cyclopropylamine (**a**) NADPH	1.57 (±0.12)	0.43 (±0.14)	1.45 (±0.01)	2.98 (±0.6)
	Propargylamine (**b**) NADPH	0.893 (±0.01)	0.103 (±0.01)	1.95 (±0.032)	2.43 (±0.36)
	Allylamine (**c**) NADPH	1.00 (±0.07)	0.33 (±0.04)	2.01 (±0.11)	3.32 (±0.46)
	Propylamine (**d**) NADPH	1.26 (±0.01)	0.64 (±0.2)	n.d.	n.d.
	Methylamine (**e**) NADPH	0.46 (±0.08)	0.32 (±0.03)	0.60 (±0.04)	2.12 (±0.43)
	Benzylamine (**f**) NADPH	0.81 (±0.02)	0.89 (±0.02)	0.10 (±0.01)	2.14 (±0.267)
	Pyrrolidine (**g**) NADPH	1.84 (±0.092)	0.64 (±0.24)	0.134 (±0.01)	2.54 (±0.48)
	Piperidine (**h**) NADPH	0.64 (±0.01)	0.89 (±0.28)	n.d.	n.d.

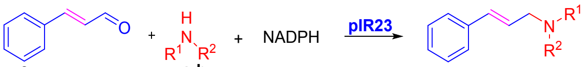

3 + a-h + NADPH → 3a-h

Varied Substrate	Saturated substrates	pIR23		*Bac*RedAm	
		k_{cat} (s^{-1})	K_m (mM)	k_{cat} (s^{-1})	K_m (mM)
cinnamaldehyde (**3**)	Cyclopropylamine (**a**) NADPH	0.36 (±0.04)	0.195 (±0.03)	0.11 (±0.01)	2.58 (±0.15)
	Propargylamine (**b**) NADPH	0.23 (0.01)	0.12 (±0.02)	0.09 (0.01)	1.96 (±0.43)
	Allylamine (**c**) NADPH	0.27 (±0.01)	0.33 (±0.05)	0.10 (±0.01)	1.89 (±0.65)
	Propylamine (**d**) NADPH	0.48 (±0.02)	0.77 (±0.09)	n.d.	n.d.
	Methylamine (**e**) NADPH	0.13 (±0.01)	0.27 (±0.07)	0.04 (±0.01)	2.54 (±0.12)
	Benzylamine (**f**) NADPH	0.15 (±0.02)	0.29 (± 0.02)	0.08 (±0.02)	2.45 (± 0.46)
	Pyrrolidine (**g**) NADPH	0.77 (±0.01)	0.25 (±0.07)	0.20 (±0.01)	3.34 (±0.63)
	Piperidine (**h**) NADPH	0.25 (±0.02)	0.34 (±0.05)	n.d.	n.d.

Activity measurements were performed in triplicate. Values in brackets represent standard deviation from the mean activity (kcat) or mean Km values. 1 unit of activity = the amount of pure enzyme required to consume 1 µmol of NADPH per min.

1.5 Determination of kinetic parameters

Specific activity (reductive aminase activity) was determined from initial rate measurements, monitoring NADPH depletion at 370 nm (ε = 2.216 mM−1 cm−1) using a Tecan infinite M200 microplate reader (Tecan Group, Switzerland). Steady-state kinetic measurements were performed with various concentrations of cinnamaldehyde (or hydrocinnamaldehyde) while the amine and NADPH concentrations were maintained at saturation. A typical reaction mixture contained aldehyde (0.05-20 mM), 80 mM amine nucleophile (added from buffer stock adjusted to pH 7.5), 0.5 mM NADPH, 1 % (v/v) DMSO and 5–10 µg of purified pIR23 or BacRedAM in a total volume of 200 µl (50 mM NaP*i* buffer, pH 7.5). The reaction was initiated by the addition of purified pIR23 or BacRedAm to the mixture. A unit of pIR23 or BacRedAm was equal to the amount of the pure enzyme required to consume 1 µmol NADPH per min. Activity measurements were performed in triplicate and kinetic constants were determined through nonlinear regression based on Michaelis–Menten kinetics (QtiPlot software).

1.6 Biotransformation for reductive amination of aldehydes

For pIR23-catalysed reductive amination, a 500 µl reaction mixture contained 12 mM D-glucose, 0.2 mg mL^{-1} GDH, 0.2 mM $NADP^{+}$, 0.3-0.6 mg mL^{-1} purified pIR23, 5 mM carbonyl compound, 10-20 mM amine nucleophile (in buffer adjusted to 7.5) and 2% (v/v) DMSO. The reaction volume was made up to 500 µl with NaP*i* buffer (50 mM, pH 7.5). Reactions were incubated at 30 °C with shaking at 250 r.p.m. for 6 h, after which they were quenched by the addition of 30 µl of 10 M NaOH and extracted twice with 500 EtoAc. The organic fractions were combined and dried over anhydrous $MgSO_4$ and analysed by GC-MS.

1.7 One-pot biotransformation for N-allylation of amines with acrylic acids

For the one-pot CAR-RedAm-mediated *N*-allylation of amines using acrylic acids to generate the corresponding allylic amines, a typical 500 uL reaction mixture contained 5 mM **1**, 2% v/v DMSO, purified *Sr*CAR (0.3mg mL^{-1}), 0.4 mg mL^{-1} purified pIR23, 12.5 mM amine (20 mM for piperidine), 6.5 mM ATP, 10 mM $MgCl_2$, 20 mM D-glucose, 0.6 mM $NADP^{+}$, 0.3 mg mL^{-1} purified GDH, in 50 mM NAP*i* buffer, pH 7.5. Reaction mixtures in 2 mL Eppendorf tube were incubated at 30 °C with 250 rpm shaking for 18 h. The reaction was then basified with NaOH to pH >12; 500 uL of EtoAc added, vigorously mixed, centrifuged (15 °C, 13 000 rpm, 5 min); and organic layer collected. The remaining aqueous layer was then acidified to pH ~2 and further extracted with EtOAc with centrifugation (15 °C, 13 000 rpm, 10 min). The organic layers were combined and dried over anhydrous $MgSO_4$ and samples were analysed by GC-MS.

1.8 GC-MS analysis

GC-MS analyses were performed on Agilent 5977A Series GC/MSD System with an Agilent 7890B Series GC coupled to Mass Selective Detector. Data analysis was performed using

GC/MSD MassHunter Data Acquisition and ChemStation Data Analysis software. A 30 m x 0.25 mm x 0.1 µm VF-5HT column (Agilent, Santa Clara, CA, USA) was used. The parameters of the method include: Inlet temperature = 240°C, detector temperature = 250°C, MS source= 230 °C, helium flow = 1.2 mL min^{-1}; oven temperature between 50 - 360°C, 20°C min^{-1} or 30 min^{-1}.

Section S2. Protein sequence of IREDs/RedAms

>*pIr23 (CfIRED)*
MKPGISVLGTGRMGSALVGAFLKQGYNVAVWNRTKSKCAPLAALGARVATTVRDAVADAEVVVVNV
NDYVTSEALLRQDDVTKGLRGKLIVQLTSGSPRQAREMAAWARQHELQYLDGAIMGTPNFIGEPGG
TILYSGPGALFEKYKPVLLVLGGNSLHVGSDVGHASALDSALLSFLWGSMFGVLQAVSVCEAEGLPL
GAYMEYVQATKPMVDGAVTDFVKRIQTGRFAGDEKTLATVE

>pIR13(*Ar*IRED]
MTEHGKTPVTVLGLGAMGTALVEALLAAGHPVTAWNRTASRAEGVAAKGASVASTVSEALAANKTVI
ACLLDYDSVHEVLDPVASGLEGRQLINLTNGTPGQAREMSAWAEELGAEYLDGGIMAVPPMIGTPGA
FIFYSGSGTVFGQARTALDTFGGVNYLGADPGLAPLHDIALLSGMYGNFIGVIQAFALVGSAGVKARE
FAPLLRGWMDAMSGFLERTAELIDDGDYERGVVSNIGMQAAAFPNLAKAAEEQGISAELLAPLQPLM
DKRVAAGHGAEDLVGVIELLKK

>pIR107 (99% sequence identity with WP_159272461.1)
MTDVSLIGLGPMGIALARALQASKFTLTVWNRTAERATPLLNQGTVLAPTALAAVQASPVVLVCVADY
PASRAILAAPGVHDALRGKVLVQLSTGTPQDARDDWAALSGVAYLDGALLATPGQIGRPDTPLFISGE
ARALAACRPLLEAIAGNIQHMGEPIGNAAAWDLATLSCMFGAMSGFFHGVRICEAEGLPVDGFAQMI
GAISPVLGEMIQAEGEAIHAGRYGEPESSMATCAGSG
RLFVKQAREAQLDAGFPDFLMGQFERALRAGLGNERLAAMVKVLRQPSQPA

>pIR114 (99% sequence identity with WP_176658404.1)
MTDVSLIGLGPMGLALARALQSKFTLTVWNRTAERAKPLLNQGTVLAPTALAAVQASPVVLVCVADY
TAWRTILAAPGVADALRGKVLVQLSTGTPQDAREDWAALGGVAYLDGALLATPGQIGRPDTPLFISG
EERALAACRPVLEAIAGNIQYMGEPIGNAAAWDLATLSCMFGAMSGFFHGVRICESEGLGVDAFSQM
IGAISPVLGEMISAEGEAIHANRYGEPESSMATCAGSGRLFVKQAREAKLDASFPDFLMGLFERSLSA
GFANERLAAMVKVMR

>BacRedAm (PZN88780.1)
MREPIVSAHTERAVESRGADRGSAVTVIGLGSMGSALAGAVLEAGYPTTVWNRTAGKAEP
LVRRGAARAATVAEAVSASPTVIACVLDYRALREILSTAGDALAGRTVVNLTNGTPTEARET
AAWVEGHGARYLDGGIMAVPEMIGGAESLVLYSGSAEAFETVEPVLRRFGSAMYLGADPG
LASLHDLALLAGMYGLFAGFLHAVALVGTEGVRATEFTSSLLIPWLQAMTATLPEAAAQIDA
GDYAATGSRLDMQAVALANIVEASRSQGIRPDLMLPIQALVERRVAKGGGGEDIAAVVEEV
RG

Section S3. Regression analysis plots from kinetic activity measurement of pIR23-catalysed reductive amination activity (representative plots presented)

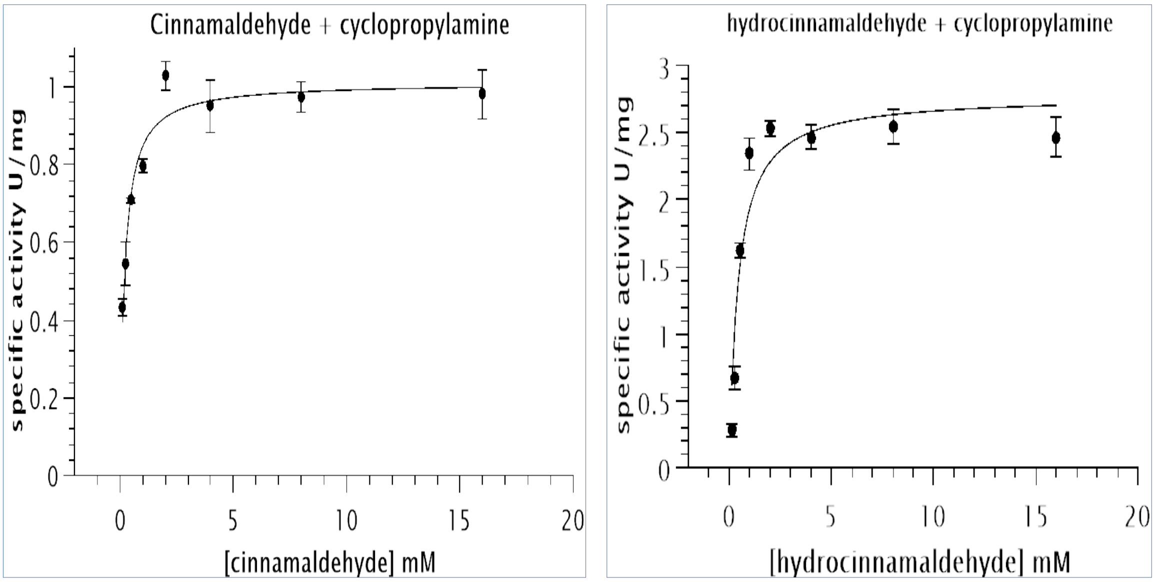

Figure S2. Plot and kinetic analysis from initial rate study of NADPH-dependent pIR23-catalysed of reductive amination of **cinnamaldehyde** (left) or **hydrocinnamaldehyde** (right) with **cyclopropylamine**. Non-linear regression analysis was performed using QTiplot software.

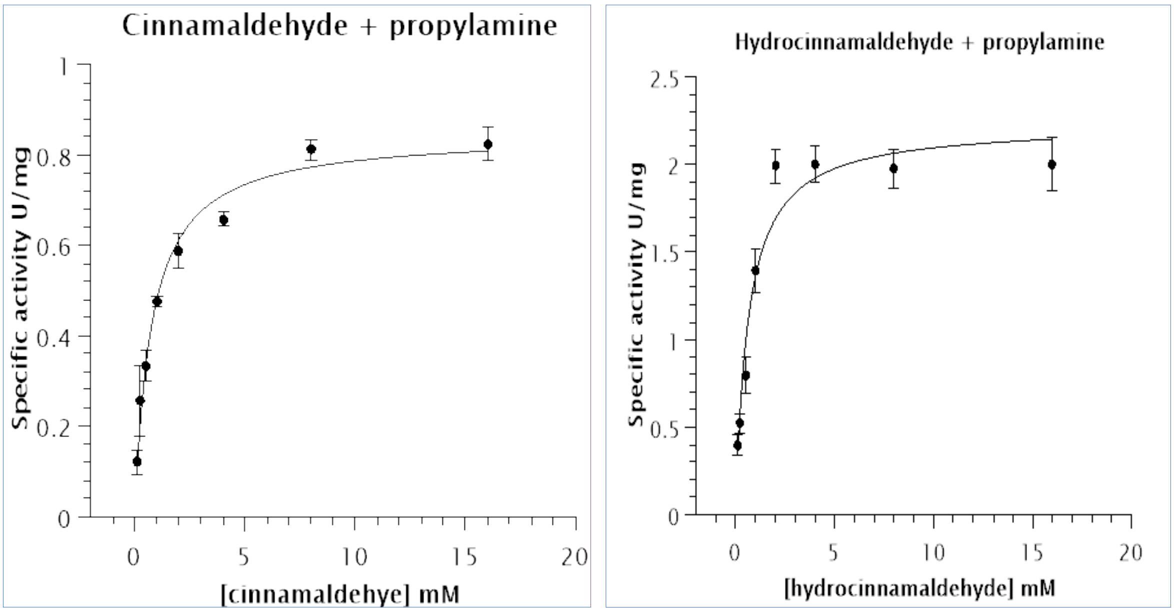

Figure S3. Plot and kinetic analysis from initial rate study of NADPH-dependent pIR23-catalysed of reductive amination of **cinnamaldehyde** (left) or **hydrocinnamaldehyde** (right) with **propylamine**. Regression analysis was performed using QTiplot software.

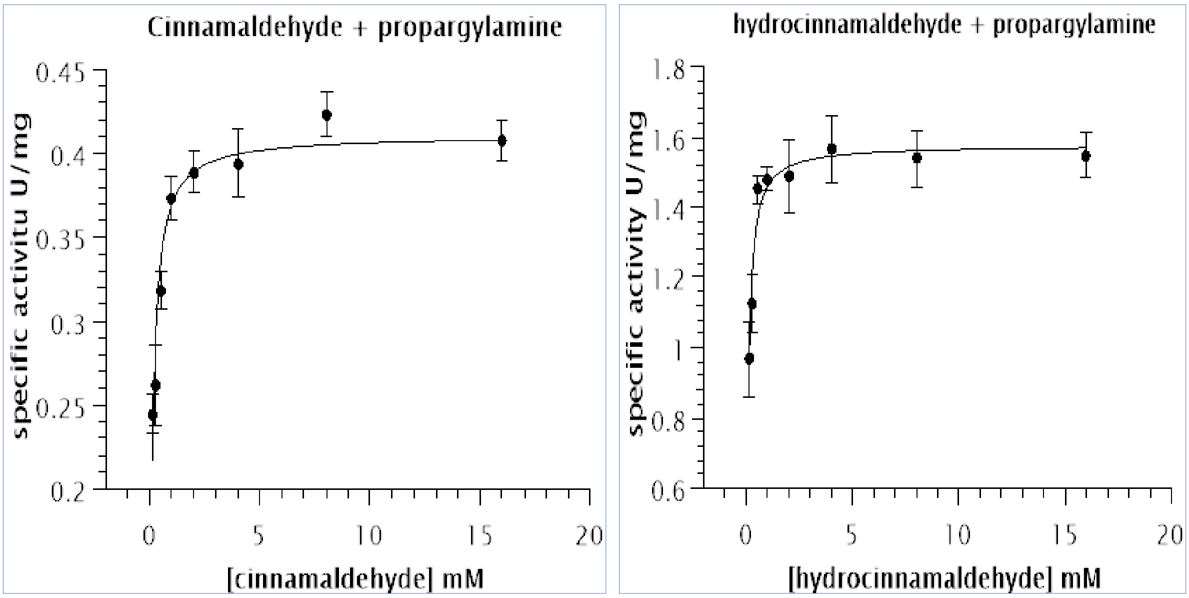

Figure S4. Plot and kinetic analysis from initial rate study of NADPH-dependent pIR23-catalysed of reductive amination of **cinnamaldehyde** (left) or **hydrocinnamaldehyde** (right) with **proparglamine**. Regression analysis was performed using QTiplot software.

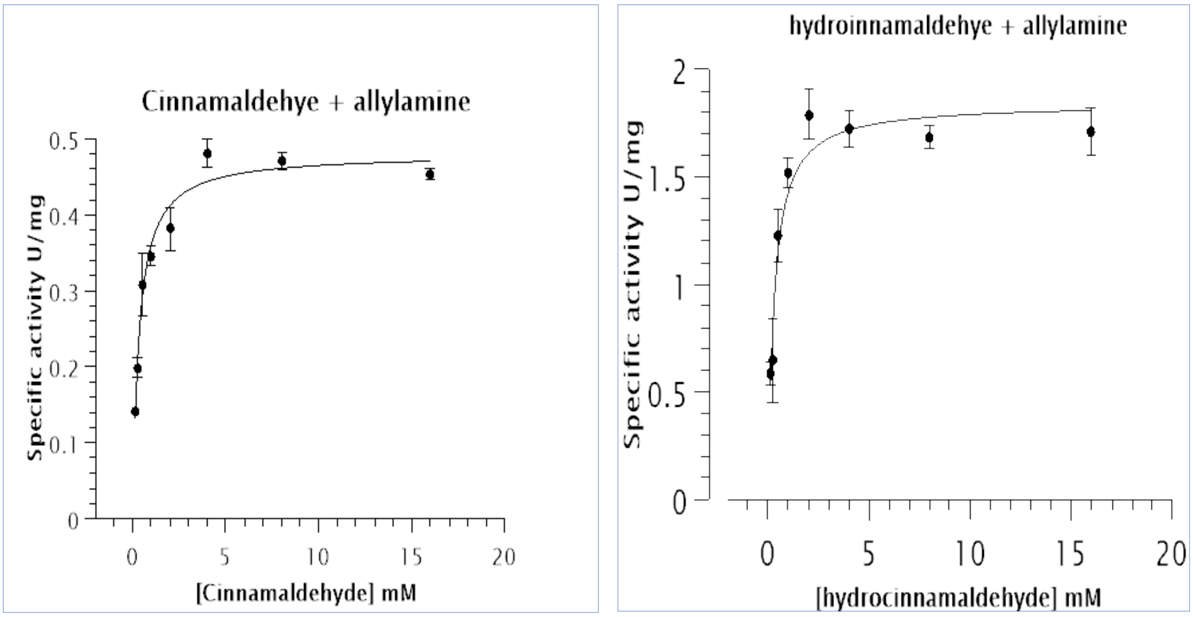

Figure S5. Plot and kinetic analysis from initial rate study of NADPH-dependent pIR23-catalysed of reductive amination of **cinnamaldehyde** (left) or **hydrocinnamaldehyde** (right) with **allylamine**. Regression analysis was performed using QTiplot software.

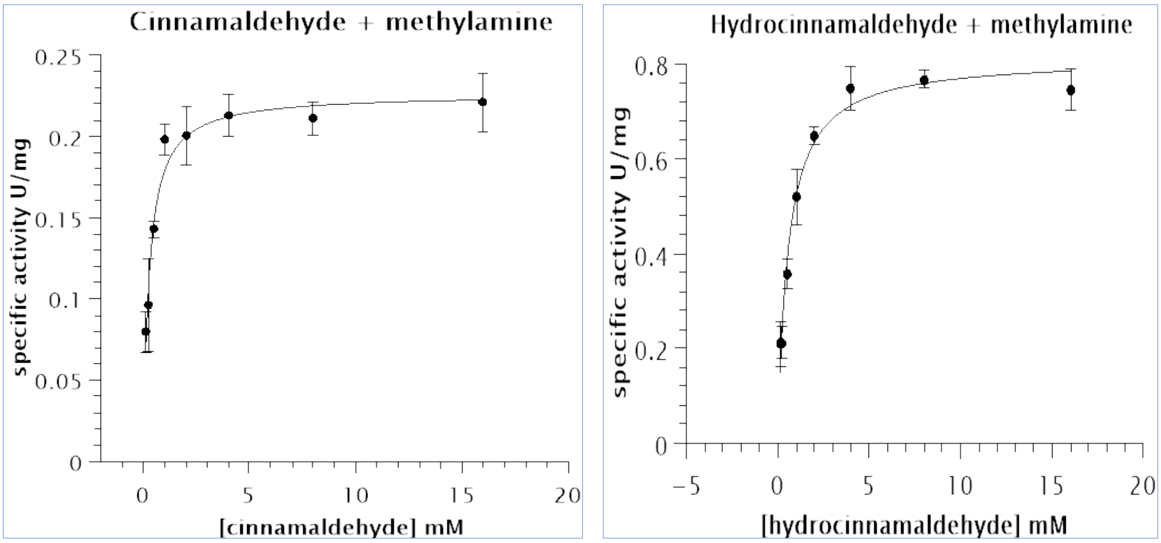

Figure S6. Plot and kinetic analysis from initial rate study of NADPH-dependent pIR23-catalysed of reductive amination of **cinnamaldehyde** (left) or **hydrocinnamaldehyde** (right) with **methylammine**. Regression analysis was performed using QTiplot software.

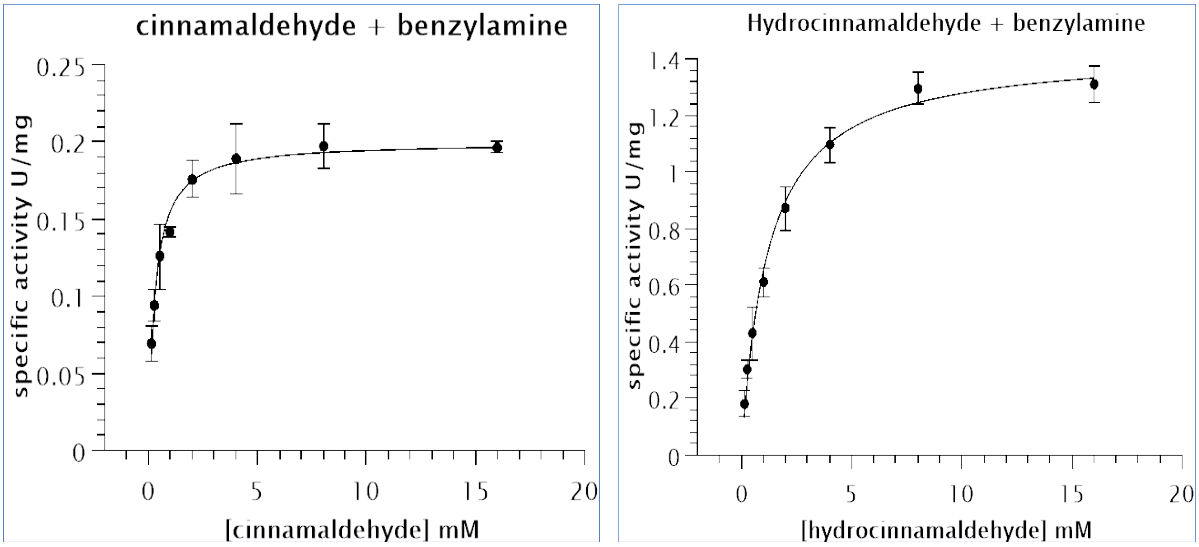

Figure S7. Plot and kinetic analysis from initial rate study of NADPH-dependent pIR23-catalysed of reductive amination of **cinnamaldehyde** (left) or **hydrocinnamaldehyde** (right) with **benzylamine**. Regression analysis was performed using QTiplot software.

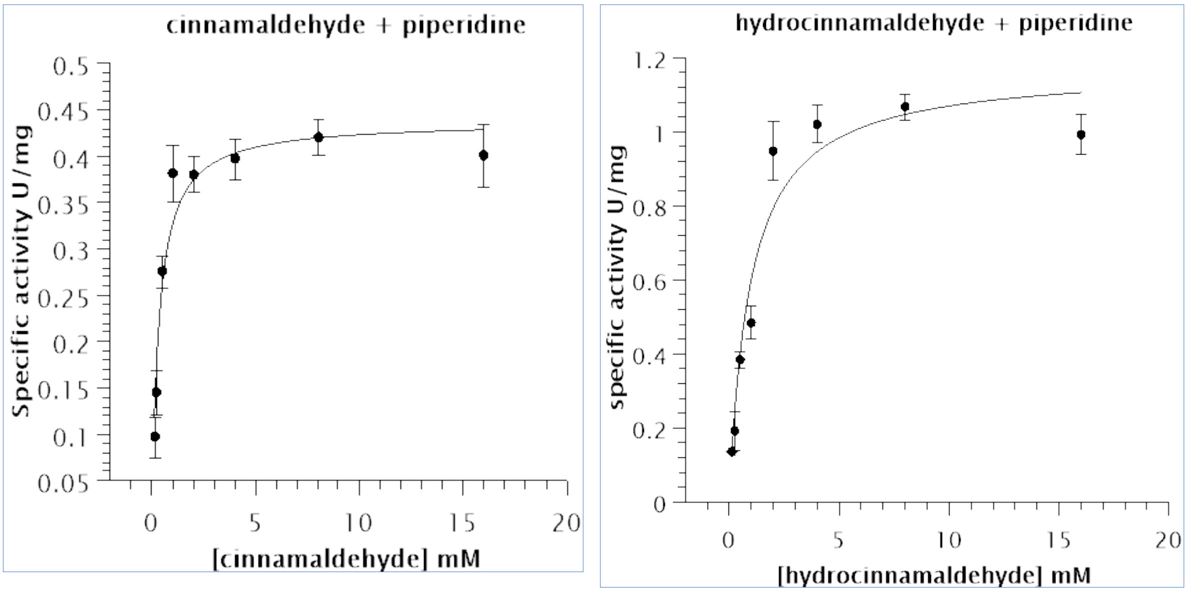

Figure S8. Plot and kinetic analysis from initial rate study of NADPH-dependent pIR23-catalysed of reductive amination of **cinnamaldehyde** (left) or **hydrocinnamaldehyde** (right) with **piperidine**. Regression analysis was performed using QTiplot software.

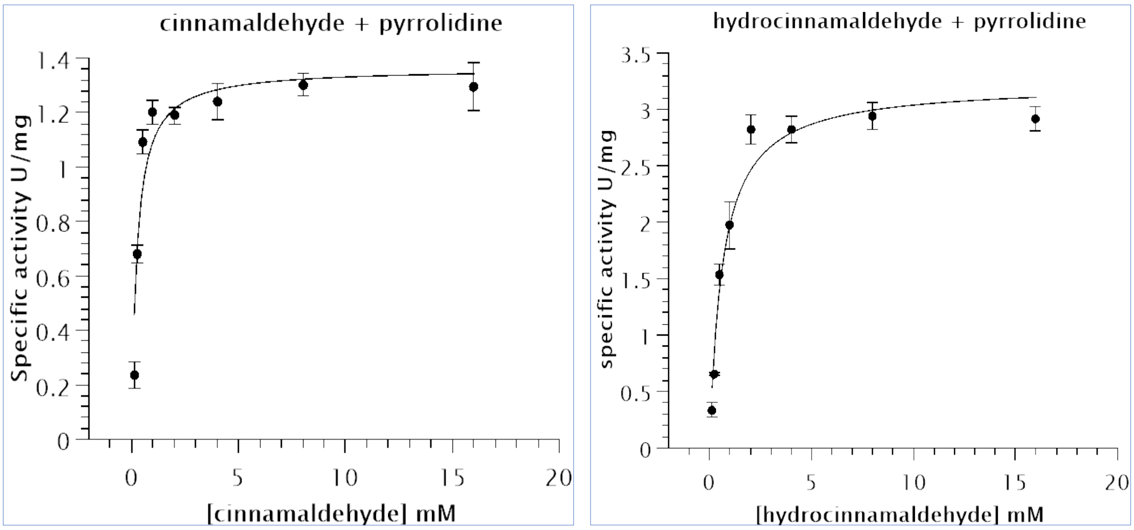

Figure S9. Plot and kinetic analysis from initial rate study of NADPH-dependent pIR23-catalysed of reductive amination of **cinnamaldehyde** (left) or **hydrocinnamaldehyde** (right) with **pyrrolidine**. Regression analysis was performed using QTiplot software.

Section S4. Analyses of GC-MS chromatograms of reductive amination reactions

Column: VF-5HT column 30 m x 0.25 mm x 0.1 µm (Agilent, Santa Clara, CA, USA). Method: inlet temperature = 240°C, detector temperature = 250°C, MS source= 230 °C, helium flow = 1.2 mL min^{-1}; oven temperature between 50 - 360°C, 20°C min^{-1}.

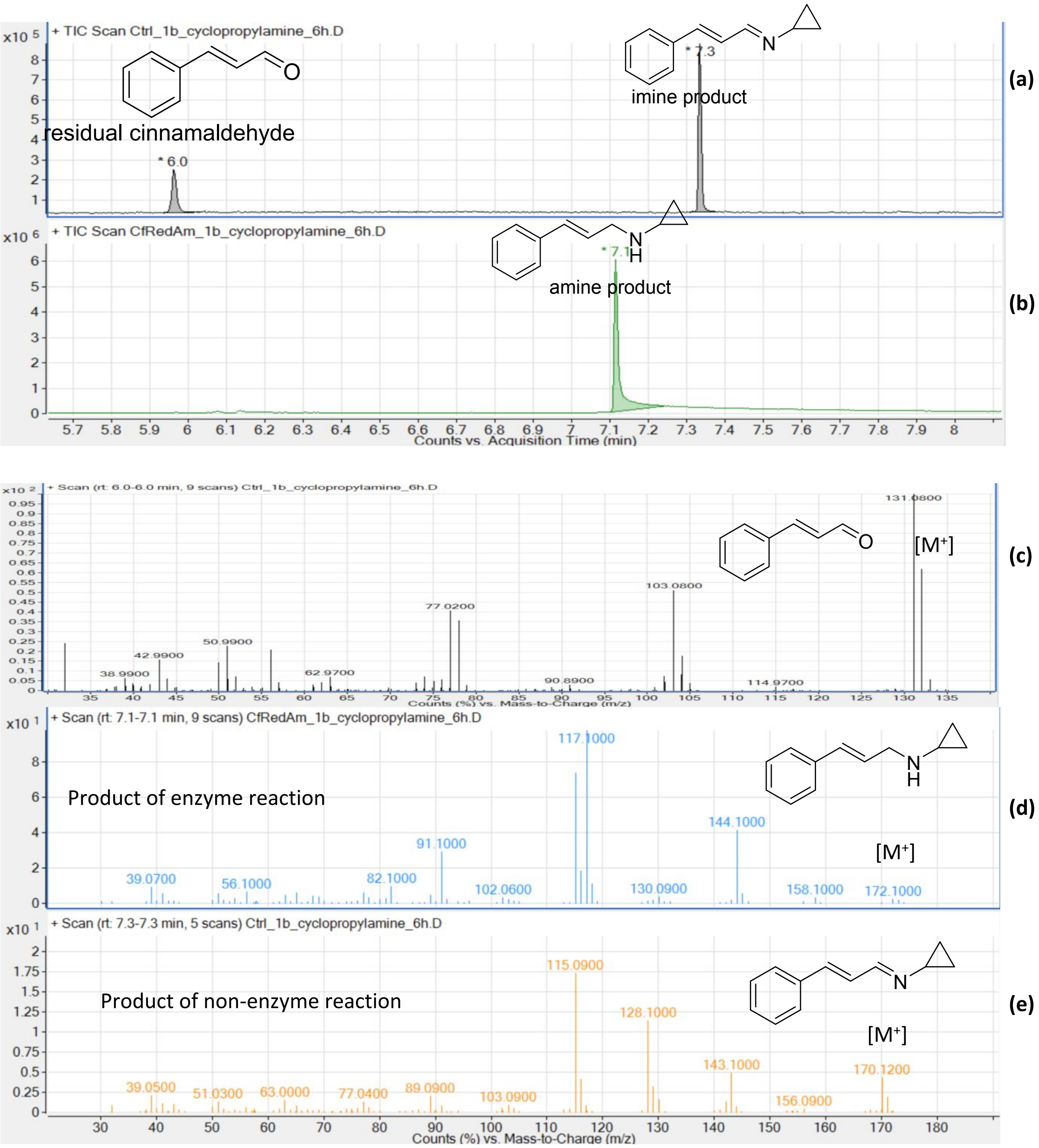

Fig. S10. GC-MS analysis of enzymatic and non-enzymatic reactions of cinnamadehyde with cyclopropylamine. **(a)** chromatogram for non-enzymatic reaction of cinnamaldehye with cyclopropylamine in phosphate buffer (pH 7.5) showing the corresponding imine product. **(b)** chromatogram for pIR23-catalysed reductive amination of cinnamaldehye with cyclopropylamine in phosphate buffer (pH 7.5) showing the corresponding amine product. (**c-e**) ESI mass spectra for residual cinnamaldehyde (from enzyme reaction), amine product (from enzyme reaction), and the imine intermediate (from the non-enzymatic reaction), respectively.

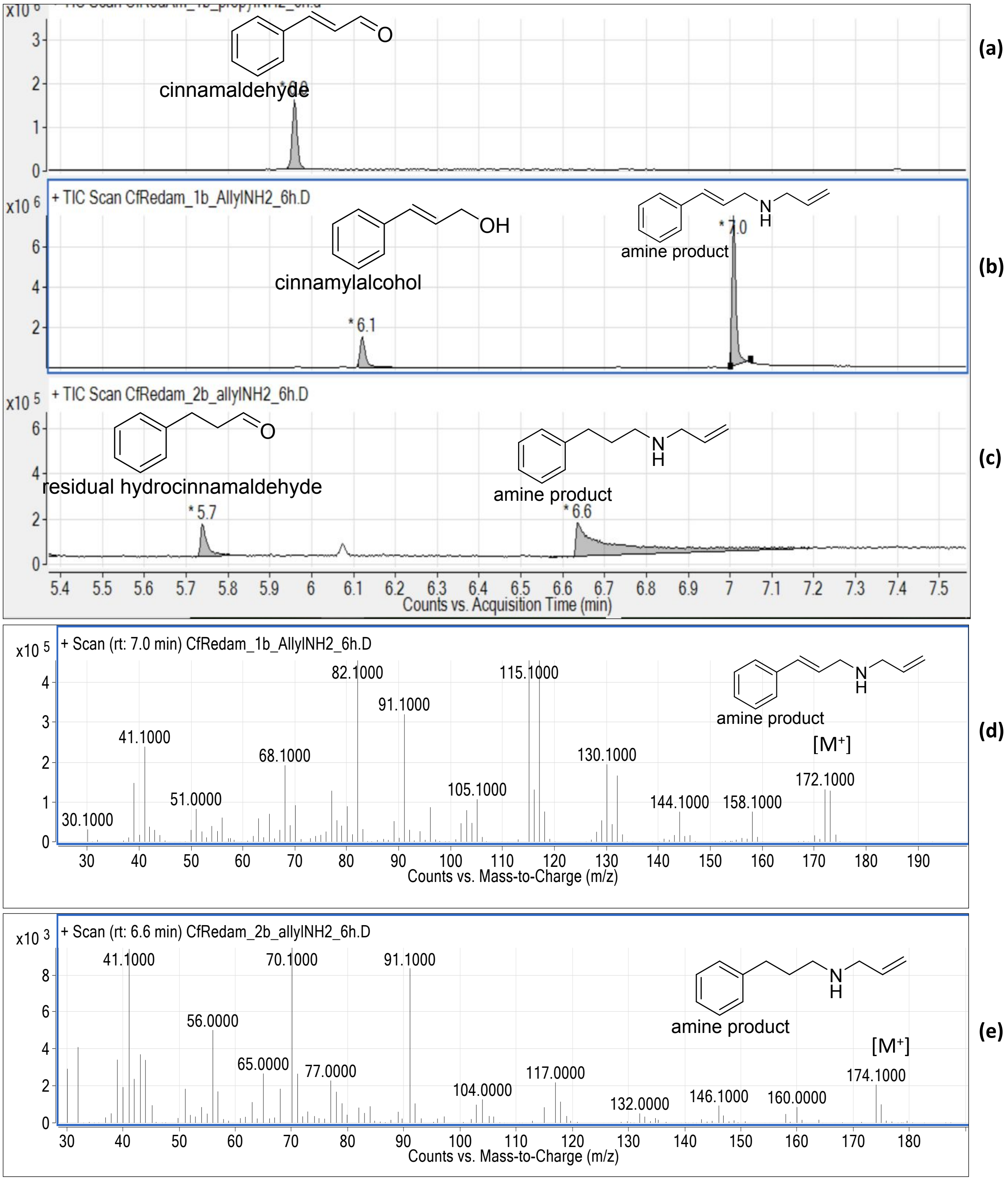

Figure S11. GC-MS analysis of biotransformation for reductive amination of cinnamadehyde/hydrocinnamaldehyde with allylamine. **(a)** chromatogram for non-enzymatic reaction of cinnamaldehye with allylamine in phosphate buffer (pH 7.5). **(b)** chromatogram for pIR23-catalysed reductive amination of cinnamaldehye with allylamine in phosphate buffer (pH 7.5) showing the corresponding amine product. **(c)** chromatogram for pIR23-catalysed reductive amination of hydrocinnamaldehye with allylamine in phosphate buffer (pH 7.5) showing the corresponding amine product. (**d & e**) ESI mass spectra of product of enzymatic biotransformation showing enzyme-catalysed biotransformation of cinnamaldehyde + allylamine, and hydrocinnamaldehyde + allylamine, respectively.

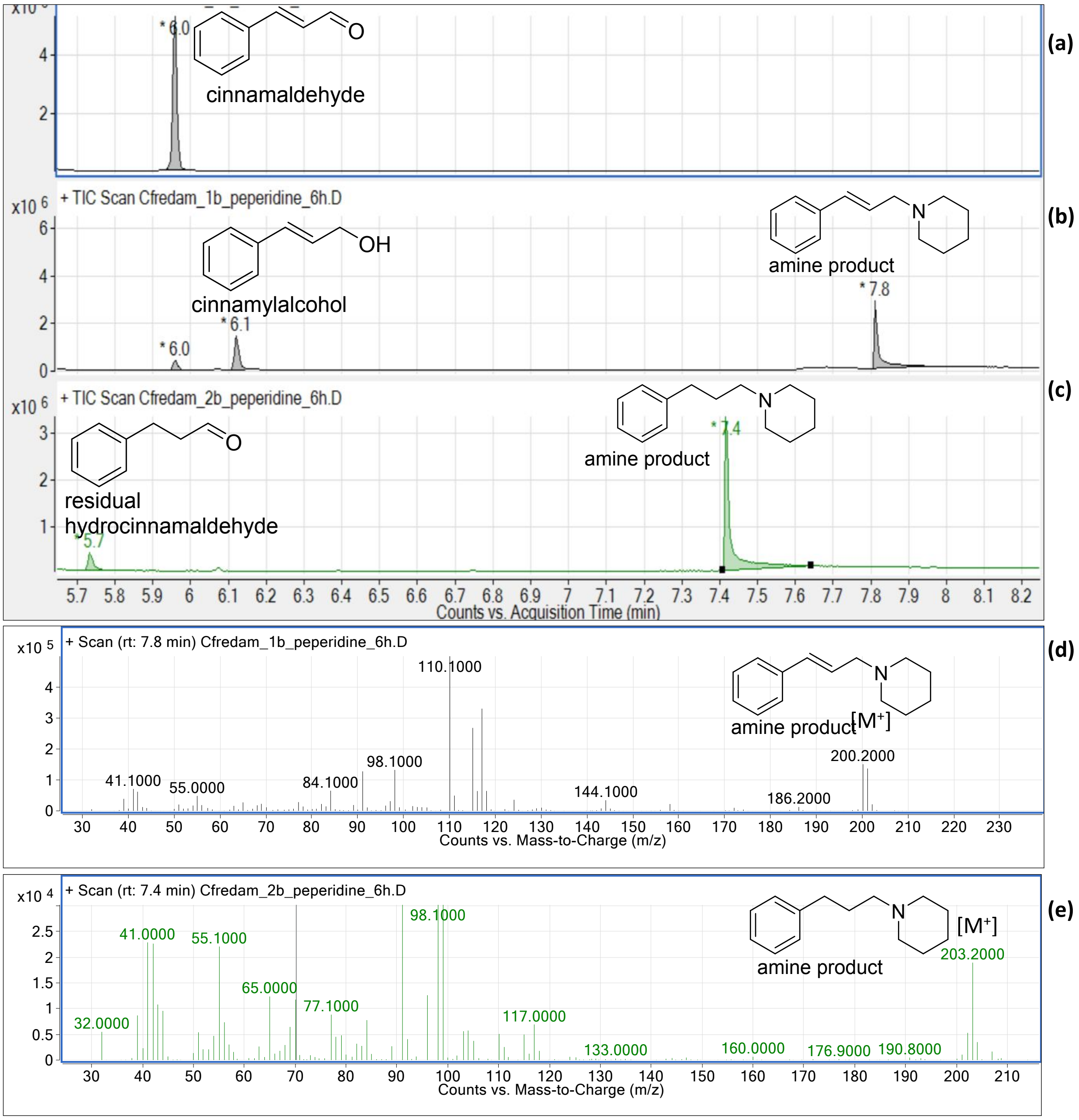

Figure S12. GC-MS analysis of biotransformation for reductive amination of cinnamadehyde/hydrocinnamaldehyde with piperidine. **(a)** chromatogram for non-enzymatic reaction of cinnamaldehye with piperidine in phosphate buffer (pH 7.5). **(b)** chromatogram for pIR23-catalysed reductive amination of cinnamaldehye with piperidine in phosphate buffer (pH 7.5) showing the corresponding amine product. **(c)** chromatogram for pIR23-catalysed reductive amination of hydrocinnamaldehye with piperidine in phosphate buffer (pH 7.5) showing the corresponding amine product. (**d** & **e**). ESI mass spectra of product of enzymatic biotransformation reactions showing enzyme-catalysed biotransformation of cinnamaldehyde + piperidine, and hydrocinnamaldehyde + piperidine, respectively.

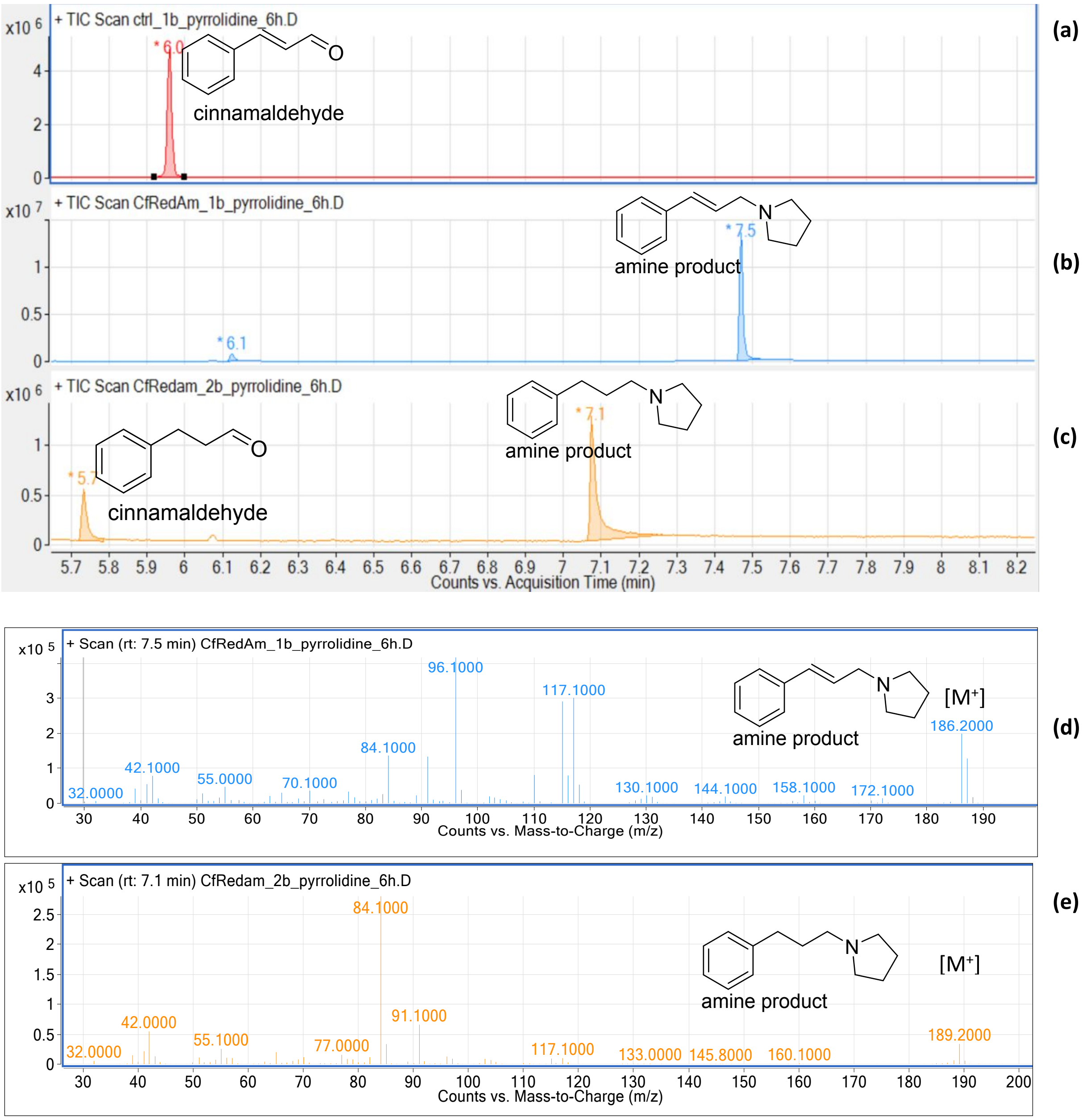

Figure S13. GC-MS analysis of biotransformation for reductive amination of cinnamadehyde/hydrocinnamaldehyde with pyrrolidine. **(a)** chromatogram for non-enzymatic reaction of cinnamaldehye with pyrrolidine in phosphate buffer (pH 7.5). **(b)** chromatogram for pIR23-catalysed reductive amination of cinnamaldehye with pyrrolidine in phosphate buffer (pH 7.5) showing the corresponding amine product. **(c)** Chromatogram for pIR23-catalysed reductive amination of hydrocinnamaldehye with pyrrolidine in phosphate buffer (pH 7.5) showing the corresponding amine product. (**d** & **e**). ESI mass spectra of product of enzymatic biotransformation reactions showing enzyme-catalysed biotransformation of cinnamaldehyde + pyrrolidine, and hydrocinnamaldehyde + pyrrolidine, respectively.

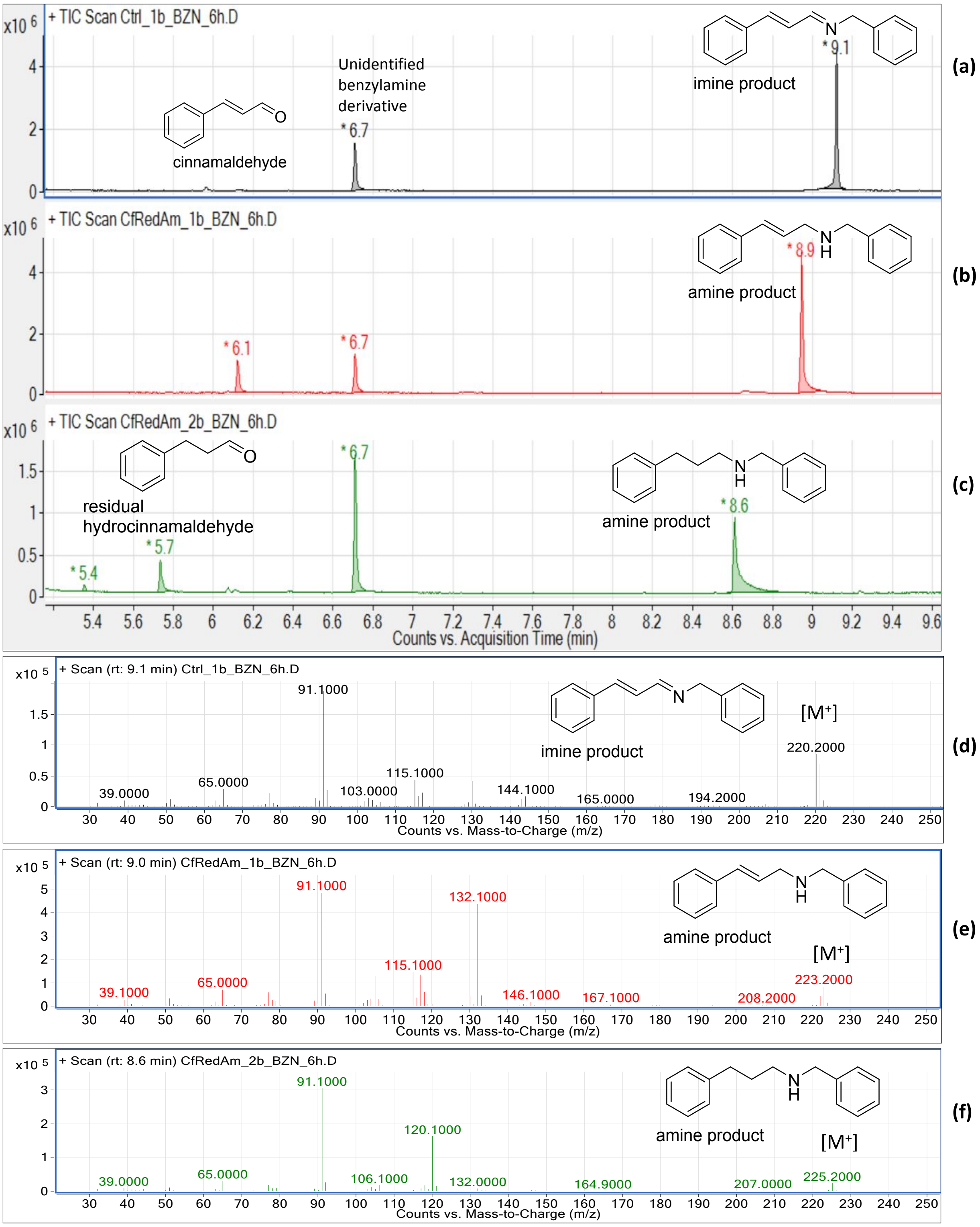

Figure S14. GC-MS analysis of biotransformation for reductive amination of cinnamadehyde/hydrocinnamaldehyde with benzylamine. **(a)** chromatogram for non-enzymatic reaction of cinnamaldehye with benzylamine in phosphate buffer (pH 7.5), showing the corresponding imine product. **(b)** chromatogram for pIR23-catalysed reductive amination of cinnamaldehye with benzylamine in phosphate buffer (pH 7.5) showing the corresponding amine product. **(c)** chromatogram for pIR23-catalysed reductive amination of hydrocinnamaldehye with benzylamine in phosphate buffer (pH 7.5) showing the corresponding amine product. (**d-f**). ESI mass spectra of products of reactions showing non-enzymatic reaction between cinnamaldehyde + benzylamine; enzyme-catalysed biotransformation of cinnamaldehyde + benzylamine, and hydrohydrocinnamaldehyde + benzylamine, respectively

Section S5. Analyses of GC-MS chromatograms one-pot reductive amination of carboxylic acids

Column: VF-5HT column 30 m x 0.25 mm x 0.1 µm (Agilent, Santa Clara, CA, USA). Method: inlet temperature = 240°C, detector temperature = 250°C, MS source= 230 °C, helium flow = 1.2 mL min^{-1}, oven temperature between 50 - 360°C, 30°C min^{-1}.

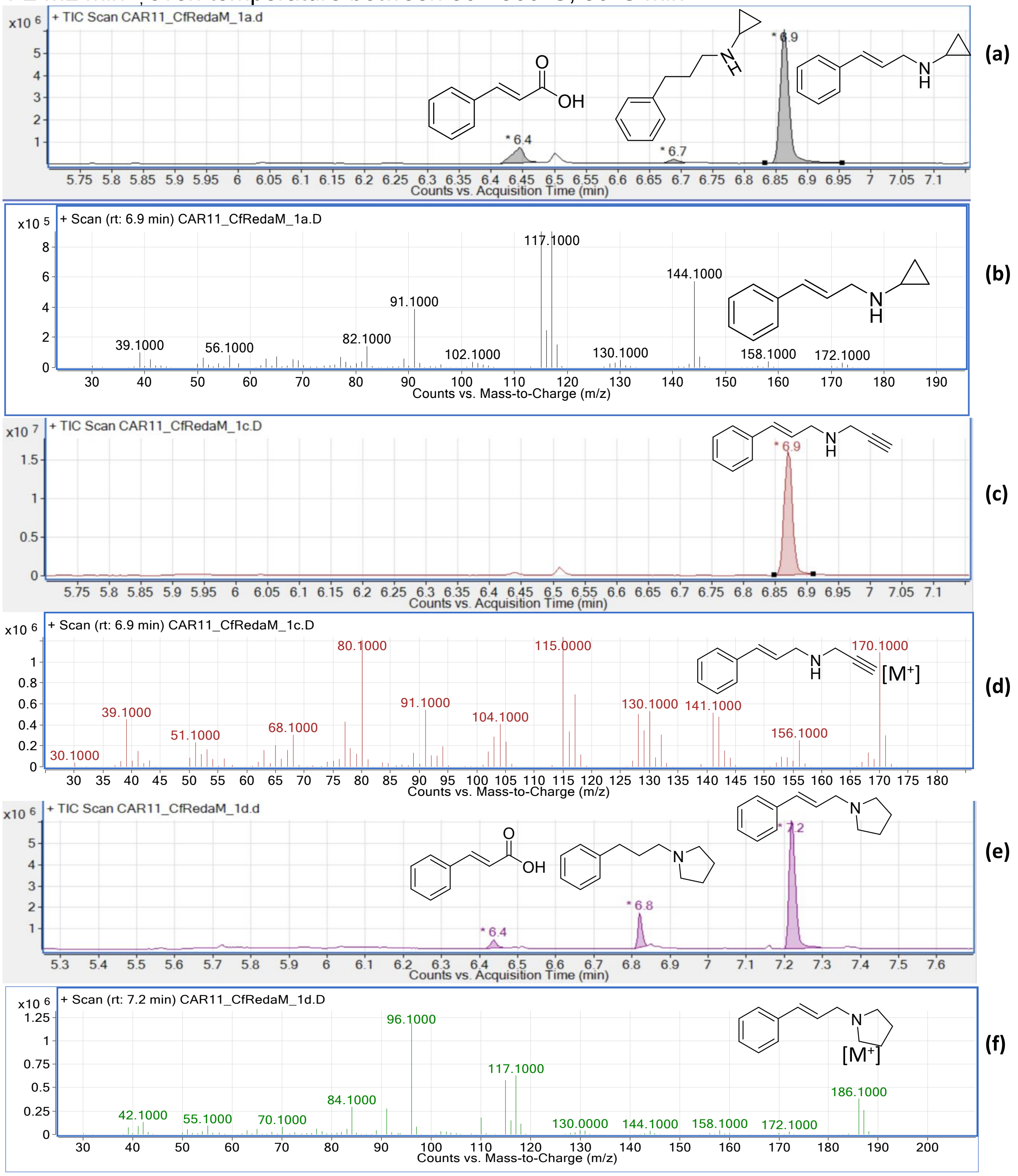

Figure S15. GC-MS analysis of one-pot biotransformation for reductive amination of cinnamic acid with different amines. **(a)** chromatogram of one-pot biotransformation for the enzyme-catalysed amination of cinnamic acid with cyclopropylamine and (**b**) the ESI mass spectra of the major product (corresponding amine). **(c)** chromatogram of one-pot biotransformation for the enzyme-catalysed amination of cinnamic acid with propargylamine and **(d)** the ESI mass spectra of the major product (corresponding amine). (**e**) chromatogram of one-pot biotransformation for the enzyme-catalysed amination of cinnamic acid with pyrrolidine and **(f)** the ESI mass spectra of the major product (corresponding amine).

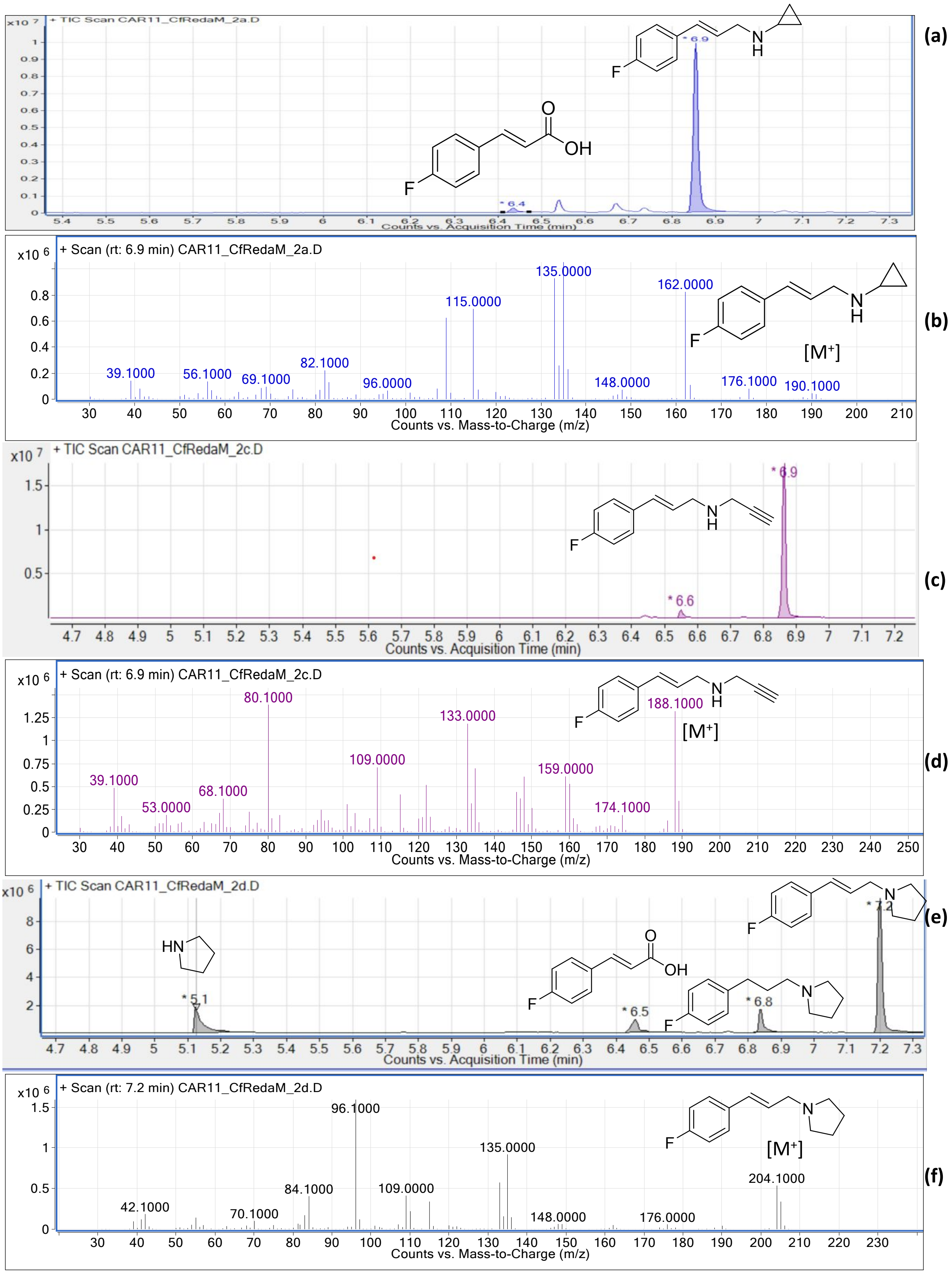

Figure S16. GC-MS analysis of one-pot biotransformation for reductive amination of *p*-fluoro cinnamic acid with different amines. **(a)** chromatogram of one-pot biotransformation for the enzyme-catalysed amination of *p*-flouro cinnamic acid with cyclopropylamine and (**b)** the ESI mass spectra of the major product (corresponding amine). **(c)** chromatogram of one-pot biotransformation for the enzyme-catalysed amination of *p*-flouro cinnamic acid with propargylamine and (**d)** the ESI mass spectra of the major product (corresponding amine). (**e**) chromatogram of one-pot biotransformation for the enzyme-catalysed amination of *p*-flouro cinnamic acid with pyrrolidine and **(f)** the ESI mass spectra of the major product (corresponding amine).

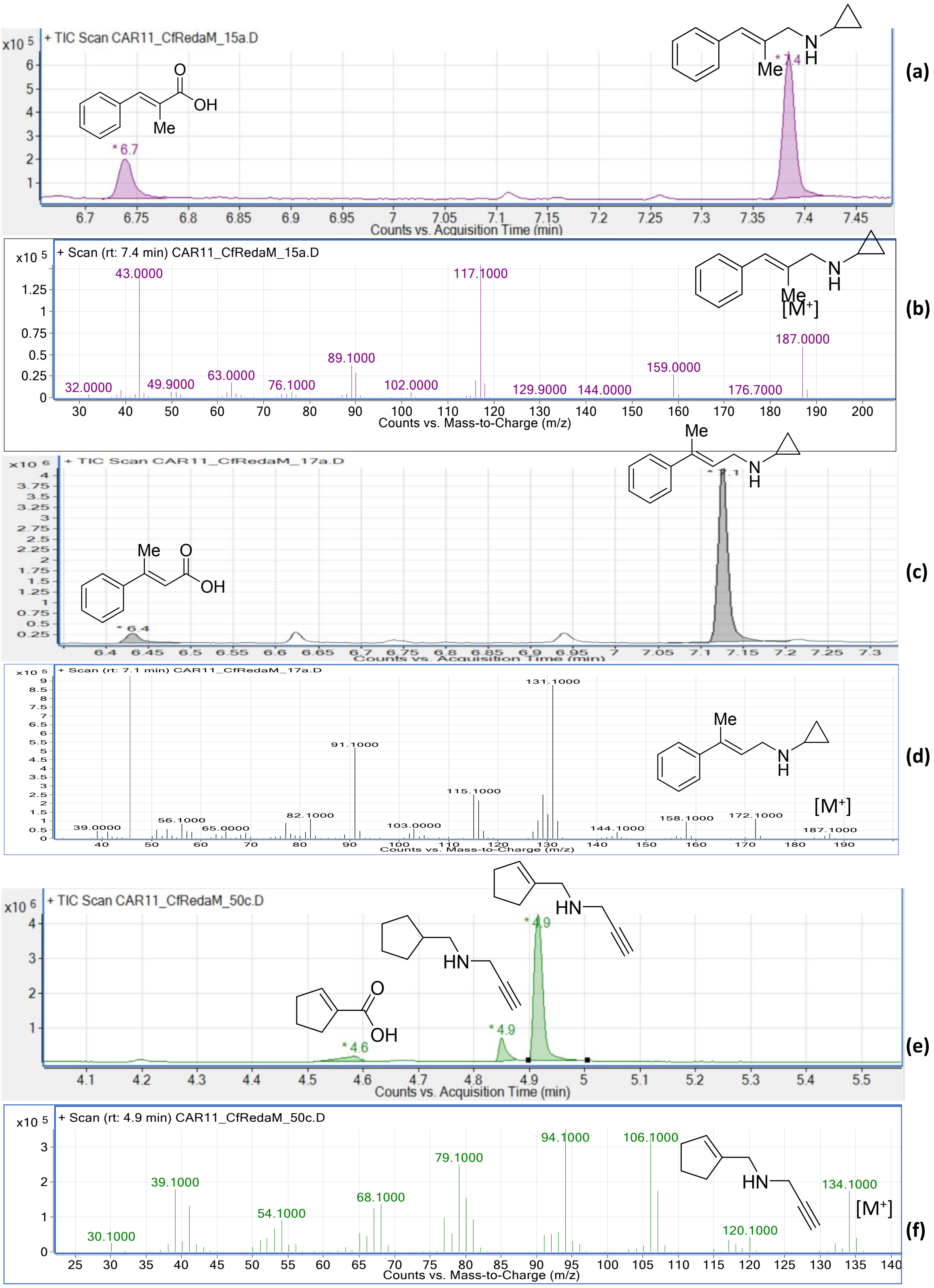

Figure S17. GC-MS analysis of one-pot biotransformation for reductive amination of alpha-methyl cinnamic acid and 1-cyclopentene-1-carboxylic acid with different amines. **(a)** chromatogram of one-pot biotransformation for the enzyme-catalysed amination of alpha-methyl cinnamic acid with cyclopropylamine and (**b**) the ESI mass spectra of the major product (corresponding amine). **(c)** chromatogram of one-pot biotransformation for the enzyme-catalysed amination of beta-methyl cinnamic acid with cyclopropylamine and (**d**) the ESI mass spectra of the major product (corresponding amine). **(e)** chromatogram of one-pot biotransformation for the enzyme-catalysed amination of 1-cyclopentene-1-carboxylic acid with propargylamine and **(f)** the ESI mass spectra of the major product (corresponding amine).

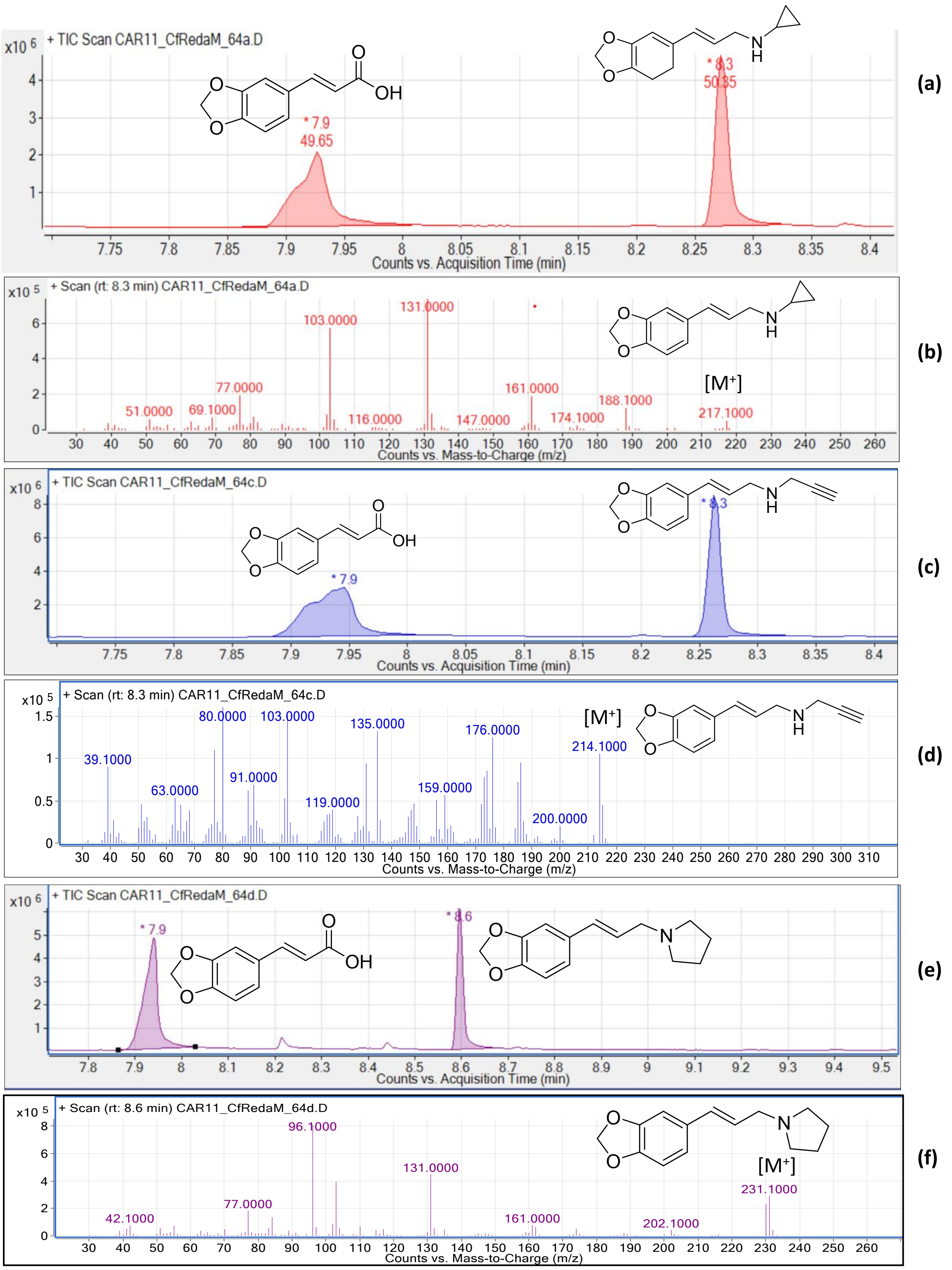

Fig. S18. GC-MS analysis of one-pot biotransformation for reductive amination of 3,4-(methylenedioxy)cinnamic acid with different amines. **(a)** chromatogram of one-pot biotransformation for the enzyme-catalysed amination of 3,4-(methylenedioxy)cinnamic acid with cyclopropylamine and (**b**) the ESI mass spectra of the major product (corresponding amine). **(c)** chromatogram of one-pot biotransformation for the enzyme-catalysed amination of 3,4-(methylenedioxy)cinnamic acid acid with propargylamine and (**d**) the ESI mass spectra of the major product (corresponding amine). (**e**) chromatogram of one-pot biotransformation for the enzyme-catalysed amination of 3,4-(methylenedioxy)cinnamic acid with pyrrolidine and **(f)** the ESI mass spectra of the major product (corresponding amine).

***Table S3**. Analysis of conserved catalytic residues among IREDs/RedAms catalysing equimolar reductive amination.*

GenBank identifier	Parent organism	Trivial name	N93	D169	Y177
XP_001827659.1	*Aspergillus oryzae*	*Asp*RedAm	N	D	Y
XP_001217087.1	*Aspergillus terreus*	AtRedam	N	D	Y
EEQ92622.1	*Ajellomyecesdermatitidis*	AdRedAm	N	D	Y
CRL25037.1	*Penicillium.camemberti*	*Pc*RedAm	N	D	S
WP_071905000.1	*Cystobacter ferrugineus*	pIR23	S	D	W
PZN88780.1	*Metagenomic Bacterium*	BacRedAm	N	D	Y
CCH32106.1	*Saccharothrix espanaensis*	GSK_IRED46	S	D	W
WP_015347361.1	*Myxococcus stipitatus*	GSK_IRED01	S	D	W
WP_010638508.1	*Streptomyces albidoflavus*	GSK_IRED10	T	D	W
WP_014351029.1	*Nocardia cyriacigeorgica*	GSK_IRED33	S	D	Y
WP_009330409.1	*Streptomyces ipomoeae*	IRED-Sip	T	D	W
WP_023809753.1	*Mesorhizobium sp.*	Pfizer_IRED44	S	D	W
WP_026324618.1	*Salinispora pacifica*	Pfizer_IRED47	T	D	W
WP_084606578.1	*Rhizobium sullae*	Pfizer_IRED48	S	D	W
WP_084831592.1	*Mesorhizobium cicero*	Pfizer_IRED50	S	D	W
WP_009737838.1	*Frankia sp.*	IRED_9	T	D	Y
WP_024839816.1	*Cellulosimicrobiumcellulans*	IRED_19	T	D	Y
Seq. available in ref. 1	*Metagenomic bacterium*	pIR107	T	D	F
Seq. available in ref. 1	*Metagenomic bacterium*	pIR114	T	D	F
Seq. available in ref. 1	*Metagenomic bacterium*	pIR238	S	D	W
Seq. available in ref. 1	*Metagenomic bacterium*	pIR241	S	D	W
Seq. available in ref. 1	*Metagenomic bacterium*	pIR355	N	D	Y
Seq. available in ref. 1	*Metagenomic bacterium*	pIR361	T	D	W

Section S6. Preparative Biotransformations

a. General procedures for preparative biotransformation reactions for the RedAm-catalysed reductive amination of cinnamaldehye with alkylamines.

Preparative scale biotransformation reactions for amination of cinnamaldehyde (100mg, 0.76 mmol, total reaction volume, 51 ml) with cyclopropylamine **a**, or propargylamine **b**, or pyrrolidine **g** were performed in 250 mL glass flasks. The 51 mL reaction mixture contained 100 mg cinnamaldehyde (15 mM), amine (2.5 equiv., 37.5 mM), D-Glucose (3.0 equiv., 45 mM), 0.6 mM $NADP^+$ and 2% v/v DMSO. The mixture was dissolved in 40 mL Tris-HCl buffer (100 mM, pH 7.8) and pH of reaction mixture was adjusted to 7.80. To this mixture was added the enzymatic components: purified pIR23 (0.4 mg mL^{-1}), and purified GDH (0.25 mg mL^{-1}) and the reaction volume was made up to 51 ml with Tris-HCl buffer (100 mM, pH 8.0). The reaction flask was sealed, incubated at 28 °C in an orbital shaker with 140 rpm shaking for 21 h. The biotransformation reactions were quenched by addition of 5 M NaOH (basified to pH ~12) and extracted twice into EtOAc (2 x 20 mL) with intermediate centrifugation (4000 r.p.m, 5min, 10 °C). The organic fractions were combined, then acidified to pH ~2 with 1 M aqueous HCl, and the phases were separated. The aqueous fraction was further basified to pH ~12 by addition of 5 M NaOH and extracted again into EtOAc (2 x 20 mL). The organic fractions were combined, dried over anhydrous MgSO4 and the solvent removed under reduced pressure to afford the corresponding allylic amine.

*b. General procedures for preparative N-allylation of amines cyclopropylamine **a**, with cinnamic acid **3i** using CAR-RedAm two-step, one-pot cascade (with supply of stoichiometric ATP)*

Preparative scale biotransformation reaction for amination of cinnamic acid **3i** (100mg, 0.68 mmol, total reaction volume 135 ml) with cyclopropylamine **a** was performed in 250 mL glass flask. The 135 mL reaction mixture contained 100 mg cinnamic acid **3i** (5 mM), cyclopropylamine **a** (2.5 equiv., 12.5 mM), D-Glucose (6.0 equiv., 30 mM), 0.6 mM $NADP^+$, 10 mM ATP, 15 mM $MgCl_2$. The mixture was dissolved in 40 mL Tris-HCl buffer (100 mM, pH 7.8) with 2% v/v DMSO added as co-solvent. The pH of reaction mixture was adjusted to 7.8. To this mixture was added the enzymatic components: purified *Sr*CAR (0.6 mg mL^{-1}), purified pIR23 (0.4 mg mL^{-1}), and GDH (0.4 mg mL^{-1}) and the reaction volume was made up to 135 ml with Tris-HCl buffer (100 mM, pH 7.8). The reaction flask was sealed, incubated at 28 °C in an orbital shaker with 140 rpm shaking for 21 h. The biotransformation reaction was quenched by addition of 5 M NaOH (basified to pH ~12) and extracted twice into EtOAc (2 x 20 mL) with

intermediate centrifugation (4000 r.p.m, 5min, 10 °C). The organic fractions were combined, then acidified to pH ~2 with 1 M aqueous HCl, and the phases were separated. The aqueous fraction was further basified to pH ~12 by addition of 5 M NaOH and extracted again into EtOAc (2 x 20 mL). The organic fractions were combined, dried over anhydrous MgSO4 and the solvent removed under reduced pressure to afford the corresponding allylic amine **3a**.

c. *General procedures for preparative N-allylation of amines cyclopropylamine **a**, propargylamine **b**, and pyrrolidine **g** with cinnamic acid **3i** or p-fluoro cinnamic acid **4** using CAR-RedAm two-step one-pot cascade (with NADPH-, and ATP-recycling systems)*

Preparative scale biotransformation reactions for amination of cinnamic acid **3i** (100mg, 0.68 mmol, total reaction volume 91ml) or *p*-fluorocinnamic acid **4** **(**100mg, 0.60, total reaction volume 80 ml) with cyclopropylamine **a**, or propargylamine **b**, or pyrrolidine **g** were performed in 250 mL glass flasks. The reaction mixture contained 100 mg cinnamic acid **3i** (7.5 mM) or p-fluorocinnamic acid **4** (7.5 mM), amine (2.5 equiv., 19 mM), D-Glucose (6.0 equiv., 45 mM), 0.5 mM $NADP^+$, 0.2 mM ATP, 30 mM $MgCl_2$, 400 mg PolyP. The mixture was dissolved in 70 mL Tris-HCl buffer (100 mM, pH 7.8) with 2% v/v DMSO added as co-solvent. The pH of reaction mixture was adjusted to 7.8. To this mixture was added the enzymatic components: purified *Sr*CAR (0.8 mg mL^{-1}), purified pIR23 (0.6 mg mL^{-1}), GDH (0.4 mg mL^{-1}), purified CHU kinase (0.6 mg/mL) and the reaction volume was made up to 91 ml (or 80 mL for **4**) with Tris-HCl buffer (100 mM, pH 7.8). The reaction flask was sealed and was incubated at 28 °C in an orbital sharker with 140 rpm shaking for 24 h. The biotransformation reaction was quenched by addition of 5 M NaOH (basified to pH ~12) and extracted twice into EtOAc (2 x 20 mL) with intermediate centrifugation (4000 r.p.m, 5min, 10 °C). The organic fractions were combined, then acidified to pH ~2 with 1 M aqueous HCl, and the phases were separated. The aqueous fraction was further basified to pH ~12 by addition of 5 M NaOH and extracted again into EtOAc (2 x 20 mL). The organic fractions were combined, dried over anhydrous MgSO4 and the solvent removed under reduced pressure to afford the corresponding allylic amine.

N-cinnamylcyclopropanamine (**3a**)

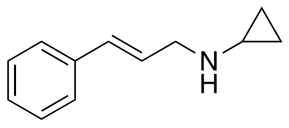

Product **3a** (pale yellow oil). **^{1}H NMR** (500 MHz, DMSO-d6) δ 7.39 (dd, *J* = 8.2, 1.4 Hz, 2H), 7.31 (t, *J* = 7.7 Hz, 2H), 7.24 – 7.17 (m, 1H), 6.53 – 6.46 (m, 1H), 6.32 (dt, *J* = 16.0, 6.1 Hz, 1H), 3.34 (dd, *J* = 6.1, 1.6 Hz, 2H), 2.31 (br s, 1H), 2.11 (tt, *J* = 6.7, 3.6 Hz, 1H), 0.35 (dt, *J* = 6.2, 3.0 Hz, 2H), 0.27 – 0.21 (m, 2H). **^{13}C NMR** (126 MHz, DMSO-d6) δ 137.06, 129.72, 129.60, 128.55, 127.08, 125.98, 50.97, 29.96, 6.12. GCMS (EI) = 172.1.

N-cinnamylpropargylamine (**3b)**

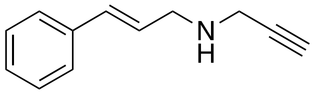

Product **3b** (yellow oil). **^{1}H NMR (**500 MHz, DMSO-d6) δ 7.43 – 7.37 (m, 2H), 7.35 – 7.28 (m, 2H), 7.25 – 7.18 (m, 1H), 6.52 (dt, *J* = 16.0, 1.6 Hz, 1H), 6.29 (dt, *J* = 15.9, 6.1 Hz, 1H), 3.36 (dd, *J* = 6.1, 1.6 Hz, 2H), 3.32 (d, *J* = 2.4 Hz, 2H), 3.06 (t, *J* = 2.4 Hz, 1H). **^{13}C NMR** (126 MHz, DMSO-d6) δ 127.22, 120.62, 119.05, 118.88, 117.52, 116.38, 73.29, 63.93, 39.82, 27.03. GCMS (EI) = 170.1.

N-cinnamylpyrrolidine (**3g**)

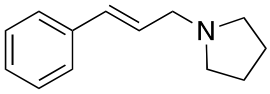

Product **3g** (pale yellow oil). **^{1}H NMR** (500 MHz, DMSO-d6) δ 7.45 – 7.39 (m, 2H), 7.33 – 7.26 (m, 2H), 7.25 – 7.19 (m, 1H), 6.52 (dt, *J* = 15.8, 1.5 Hz, 1H), 6.33 (dt, *J* = 15.9, 6.5 Hz, 1H), 3.49 (br s, 1H), 3.18 (dd, *J* = 6.5, 1.5 Hz, 2H), 2.45 (ddt, *J* = 7.4, 5.8, 2.2 Hz, 4H), 1.77 – 1.54 (m, 4H). ^{13}C NMR (126 MHz, DMSO-d6) δ 136.49, 130.44, 128.20, 127.92, 127.86, 126.91, 125.78, 57.24, 53.19, 53.09, 22.83. GCMS (EI) = 186.1.

(*E*)-*N*-(3-(4-fluorophenyl)allyl)cyclopropanamine (**4a**)

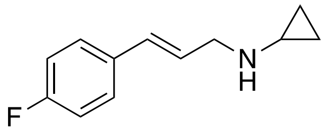

Product **4a** (pale yellow oil). **^{1}H NMR** (500 MHz, DMSO-d6) δ 7.48 – 7.39 (m, 2H), 7.16 – 7.10 (m, 2H), 6.49 (dt, *J* = 16.0, 1.6 Hz, 1H), 6.27 (dt, *J* = 16.0, 6.1 Hz, 1H), 3.32 (dd, *J* = 6.2, 1.6 Hz, 3H), 2.11 (tt, *J* = 6.7, 3.6 Hz, 1H), 0.35 (td, *J* = 6.3, 4.1 Hz, 2H), 0.25 – 0.21 (m, 2H). **^{13}C NMR** (126 MHz, DMSO-d6) δ 162.78, 160.84, 134.12, 134.09, 130.13, 130.11, 128.89, 128.30, 128.24, 115.91, 115.74, 51.39, 30.43, 9.33, 6.58, 6.03. GCMS (EI) = 190.1.

Section 7. NMR spectra of products isolated form CAR-RedAm one-pot cascade for the amination of cinnamic acids with alkylamines

N-cinnamylcyclopropanamine (**3a**)

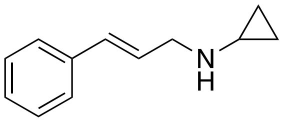

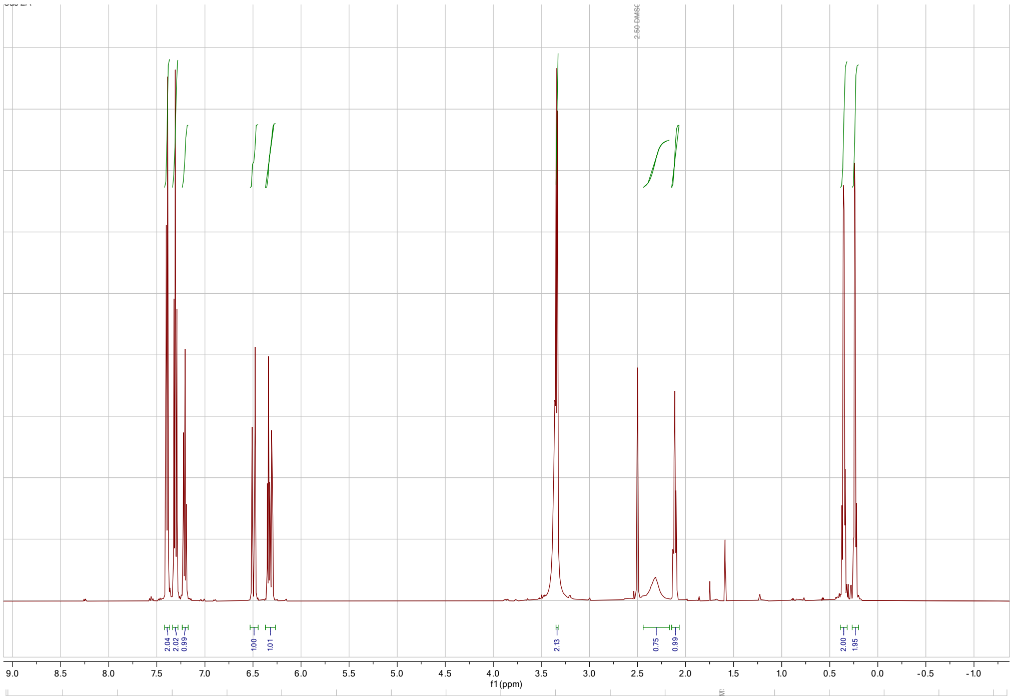

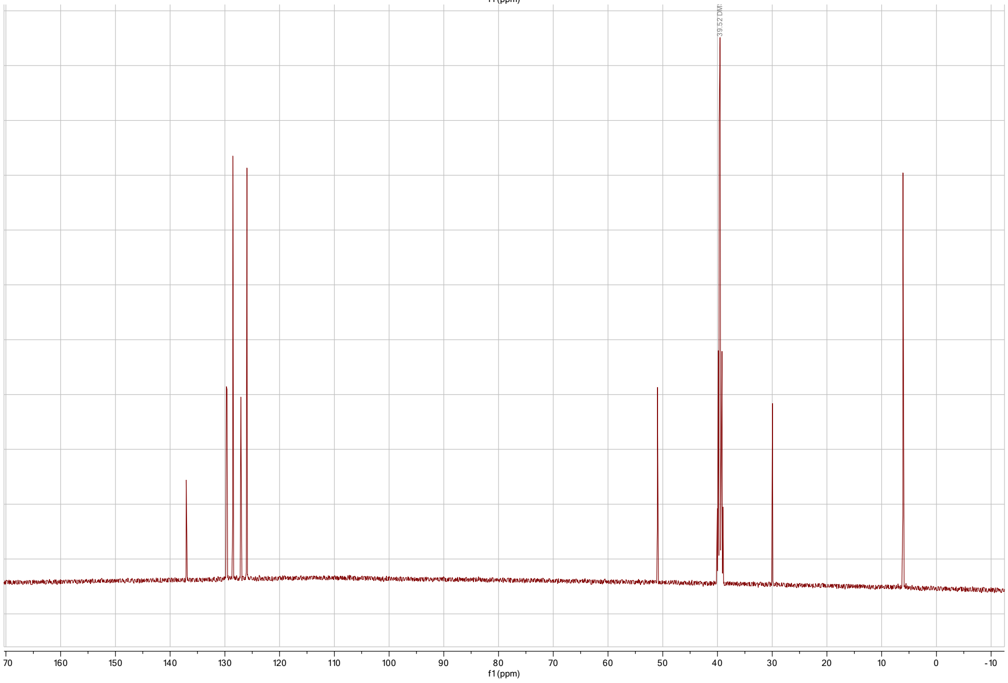

N-cinnamylpropargylamine (**3b**)

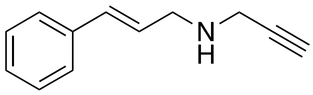

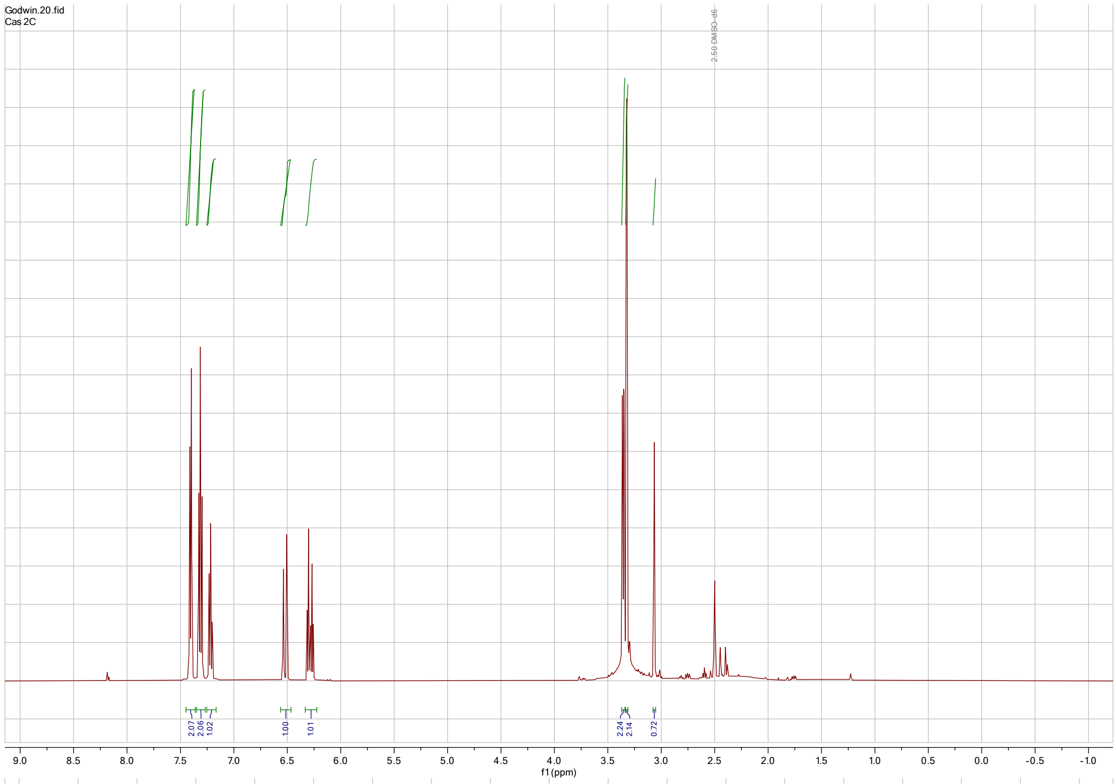

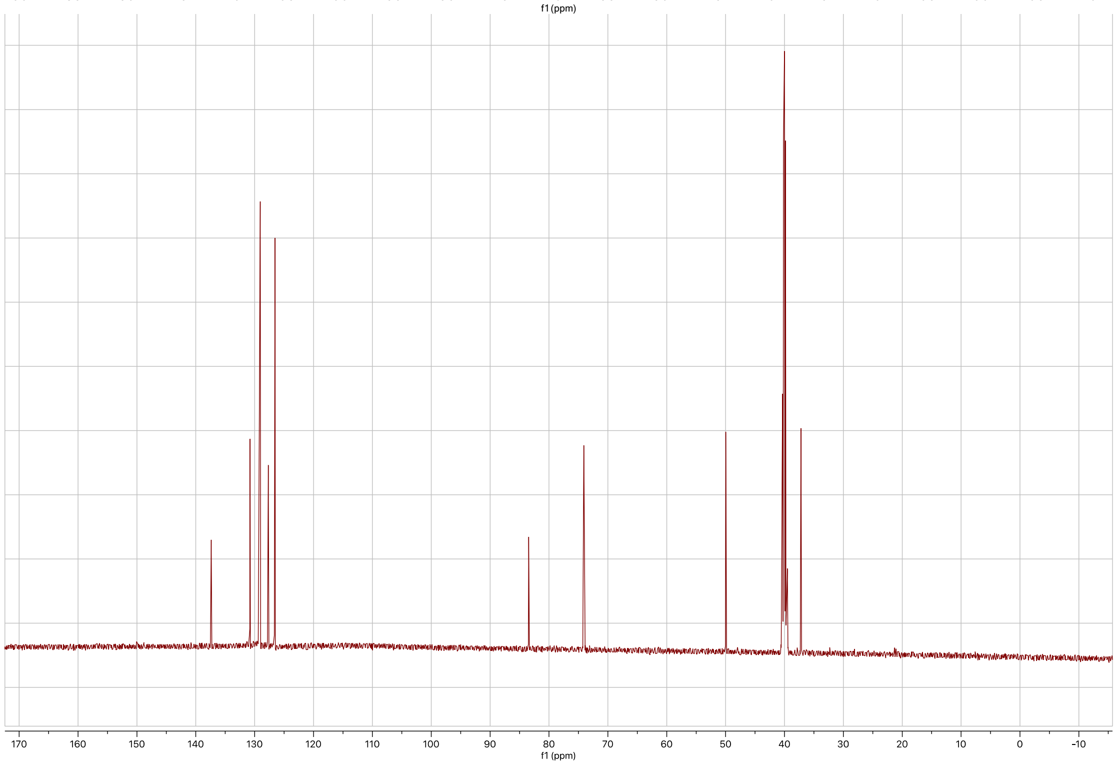

N-cinnamylpyrrolidine (**3g**)

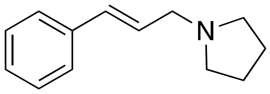

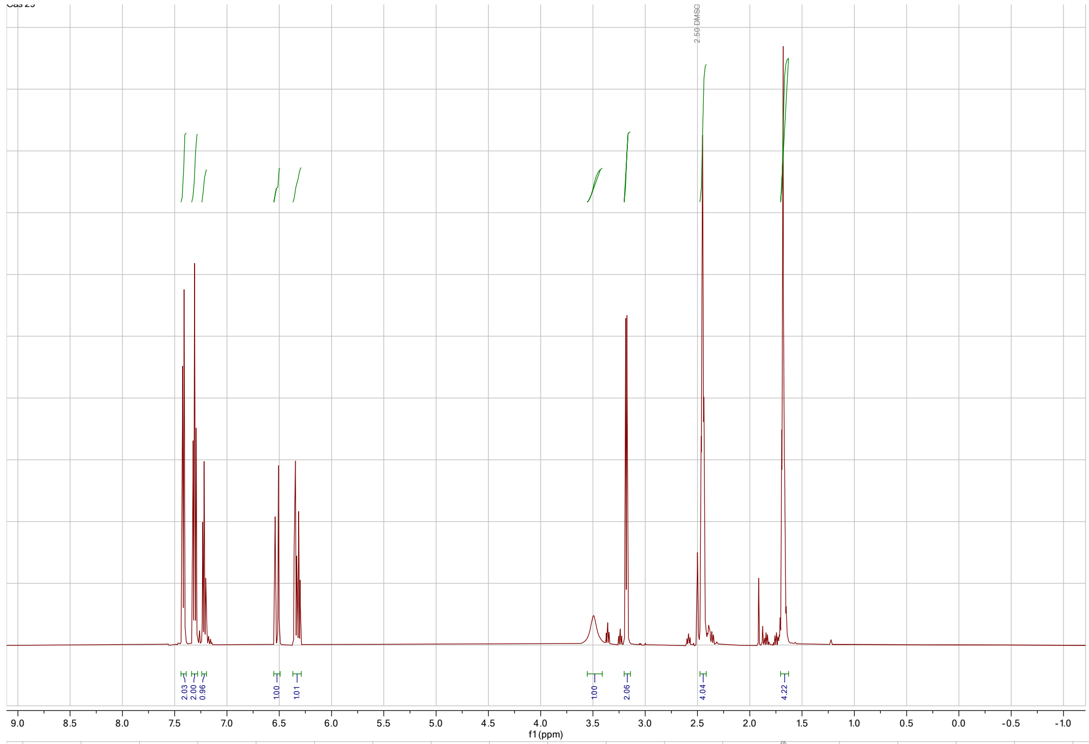

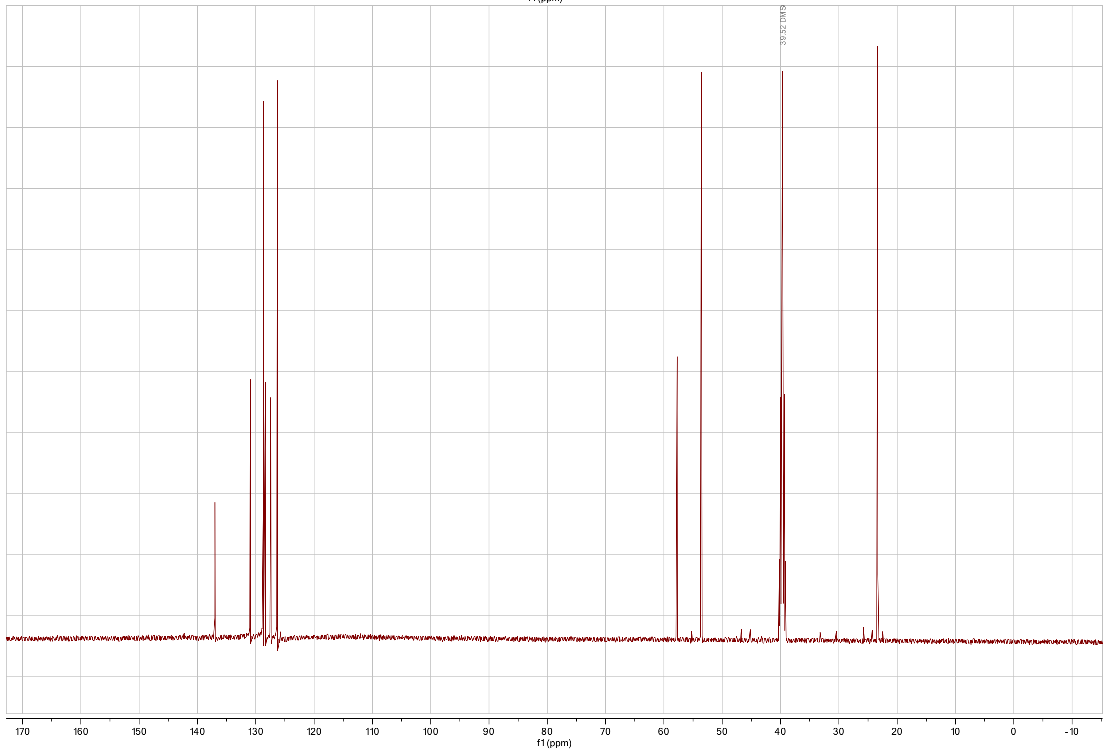

(*E*)-*N*-(3-(4-fluorophenyl)allyl)cyclopropanamine (**4a**)

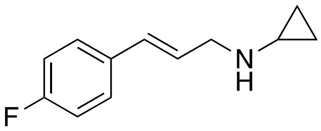

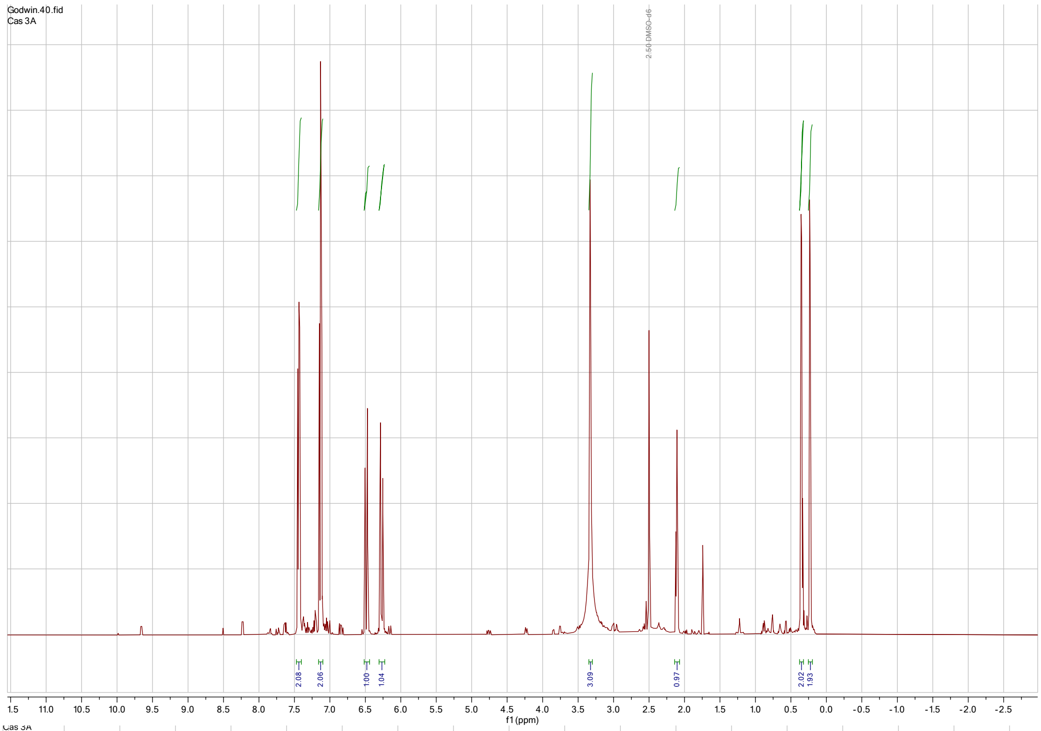

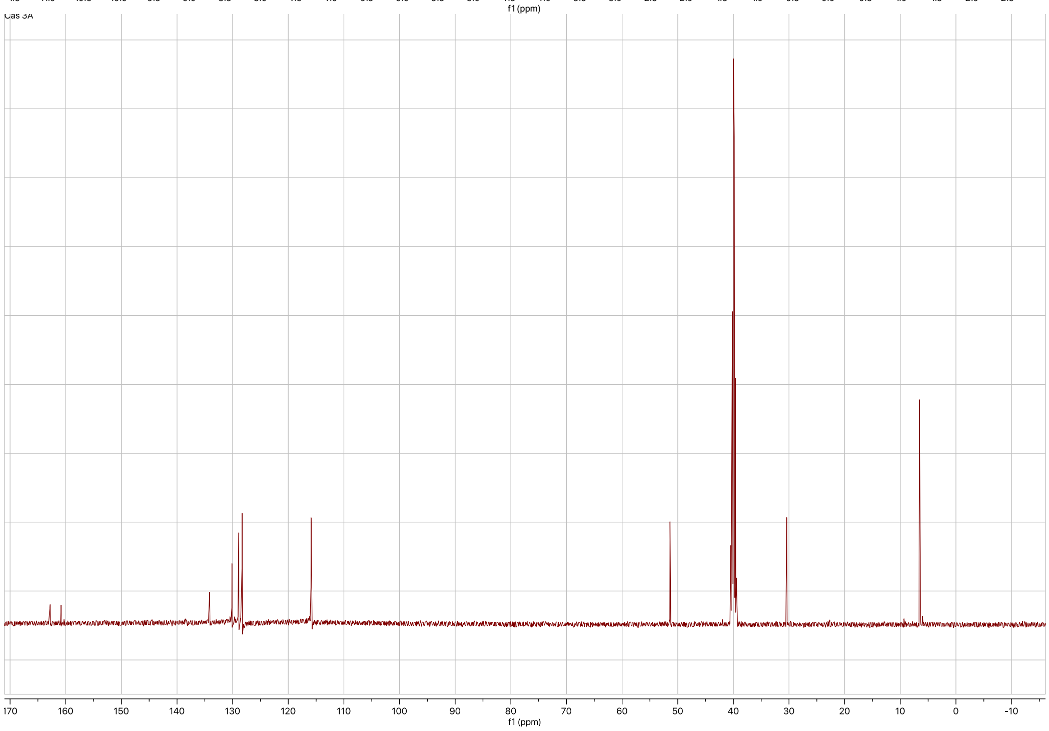

References

[1] D. Gahloth, M. S. Dunstan, D. Quaglia, E. Klumbys, M. P. Lockhart-Cairns, A. M. Hill, S. R. Derrington, N. S. Scrutton, N. J. Turner, D. Leys, *Nature Chemical Biology* **2017**, *13*, 975–981.
[2] G. A. Aleku, G. W. Roberts, D. Leys, *Green Chem.* **2020**, *22*, 3927–3939.
[3] B. P. Nocek, A. N. Khusnutdinova, M. Ruszkowski, R. Flick, M. Burda, K. Batyrova, G. Brown, A. Mucha, A. Joachimiak, Ł. Berlicki, A. F. Yakunin, *ACS Catal.* **2018**, *8*, 10746–10760.
[4] J. R. Marshall, P. Yao, S. L. Montgomery, J. D. Finnigan, T. W. Thorpe, R. B. Palmer, J. Mangas-Sanchez, R. A. M. Duncan, R. S. Heath, K. M. Graham, D. J. Cook, S. J. Charnock, N. J. Turner, *Nature Chemistry* **2020**, 1–9.
[5] G. A. Aleku, S. P. France, H. Man, J. Mangas-Sanchez, S. L. Montgomery, M. Sharma, F. Leipold, S. Hussain, G. Grogan, N. J. Turner, *Nature Chemistry* **2017**, *9*, 961–969.
[6] R. C. Edgar, *Nucleic Acids Res* **2004**, *32*, 1792–1797.
